# Heterotypic tumor spheroids: a platform for nanomedicine evaluation

**DOI:** 10.1186/s12951-023-02021-y

**Published:** 2023-08-02

**Authors:** Faezeh Vakhshiteh, Zeinab Bagheri, Marziye Soleimani, Akram Ahvaraki, Parisa Pournemat, Seyed Ebrahim Alavi, Zahra Madjd

**Affiliations:** 1 Oncopathology Research Center, Iran University of Medical Sciences (IUMS), Tehran, Iran; 2 Department of Cell and Molecular Biology, Faculty of Life Sciences and Biotechnology, Shahid Beheshti University, Tehran, 1983969411 Iran; 3 Faculty of Medicine, Frazer Institute, The University of Queensland, Brisbane, QLD 4102 Australia; 4 Department of Molecular Medicine, Faculty of Advanced Technologies in Medicine, Iran University of Medical Sciences (IUMS), Tehran, Iran

**Keywords:** Heterotypic spheroid, Co-culture spheroids, Nanomedicine, Fibroblast, Endothelial, Immune cells, Tumor microenvironment

## Abstract

Nanomedicine has emerged as a promising therapeutic approach, but its translation to the clinic has been hindered by the lack of cellular models to anticipate how tumor cells will respond to therapy. Three-dimensional (3D) cell culture models are thought to more accurately recapitulate key features of primary tumors than two-dimensional (2D) cultures. Heterotypic 3D tumor spheroids, composed of multiple cell types, have become more popular than homotypic spheroids, which consist of a single cell type, as a superior model for mimicking in vivo tumor heterogeneity and physiology. The stromal interactions demonstrated in heterotypic 3D tumor spheroids can affect various aspects, including response to therapy, cancer progression, nanomedicine penetration, and drug resistance. Accordingly, to design more effective anticancer nanomedicinal therapeutics, not only tumor cells but also stromal cells (e.g., fibroblasts and immune cells) should be considered to create a more physiologically relevant in vivo microenvironment. This review aims to demonstrate current knowledge of heterotypic 3D tumor spheroids in cancer research, to illustrate current advances in utilizing these tumor models as a novel and versatile platform for in vitro evaluation of nanomedicine-based therapeutics in cancer research, and to discuss challenges, guidelines, and future directions in this field.

**Graphical Abstract:**

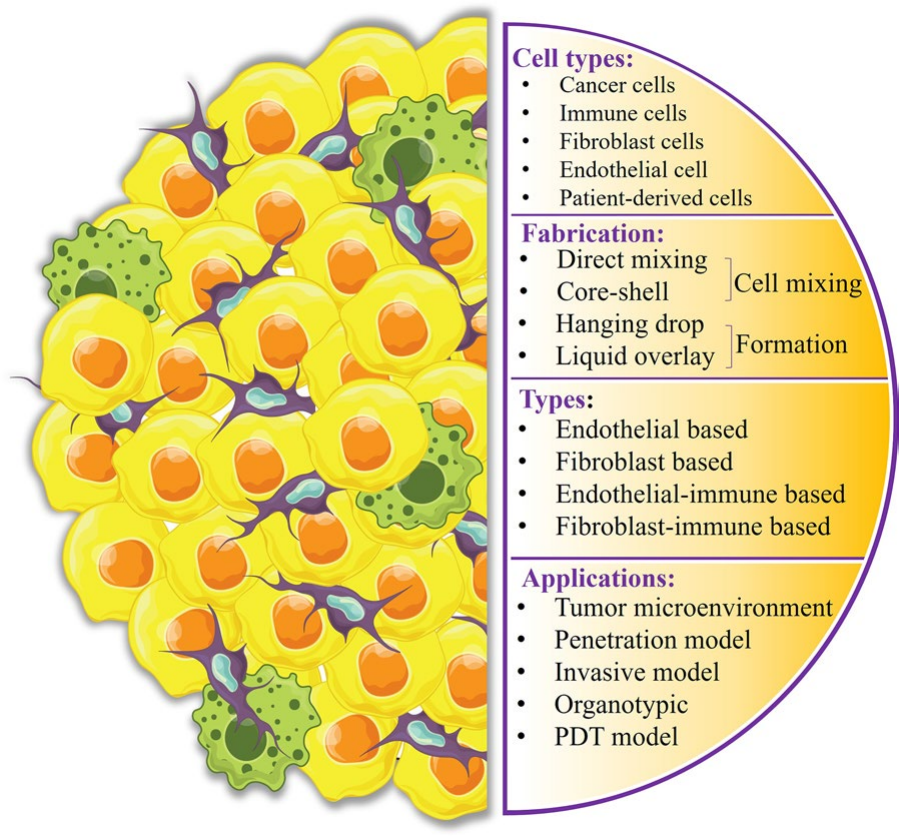

## Introduction

Nanomedicine is a developing methodology that applies nanoscience to diagnostics and therapeutics [1]. In recent years, nanoparticle-based therapeutics have been extensively investigated and have become an area of primary importance [2, 3]. Over the last 20 years, around 80 nanomedicine products have been approved by the Food and Drug Administration (FDA) and the European Medicines Agency (EMA) for marketing. The overall progress in the field of nanoscience has made significant contributions to disease diagnosis and therapy and may be beneficial in the development of patient-specific treatments [4]. Manufacturing parent drugs into nanoformulations leads to modifications in pharmacokinetics, biochemical, electromagnetic, and optical properties, resulting in changes in the toxicity profile and better treatment outcomes. Therefore, it is essential to determine the drug characteristics and toxicity profile of novel nanoparticle (NP)-based formulations in appropriate cell cultures and animal models to understand the differential features [5]. Cytotoxicity-related analyses in cell cultures are considered suitable tools for in vitro nanotoxicity evaluation [6]. However, the main in vitro NP assessment is performed on two-dimensional (2D) monolayer cultures, although various obstacles impact the effectiveness of nanomedicine for targeted tumor delivery, which are not adequately represented by 2D monocultures [7, 8]. Hence, three-dimensional (3D) spheroids have become popular over routine 2D monocultures and the importance of using 3D cell culture models, which are superior models for mimicking in vivo tumor heterogeneity/physiology, has been acknowledged.

2D cell culture systems are simple, low-cost, and well-suited for automated high-throughput drug screening, and they have been effectively used to determine several clinically relevant anticancer agents. However, 2D tissue culture conditions cannot represent the in vivo features of tumors due to their rigidity and lack of 3D structure. Therefore, drug candidates identified from 2D cultures often show unsuccessful results in clinical trials [9]. Spheroids cultured in 3D conditions have been applied as an appreciated system to evaluate various nanocarriers regarding physical/chemical characteristics, such as shape, size, surface features, and chemical composition that exhibit vital effects on tumor penetration and therapeutic effectiveness [10]. 3D spheroid models can simulate the complexity of the microenvironment in solid tumors and can replicate many features of tumors, such as hypoxic conditions, extracellular matrix (ECM) communications, pH, nutrient access, and drug permeability, which particularly affect overall toxicity [11]. The 3D culture models enable comparable topography, gene expression, metabolism, and signaling of tumor cells to that in the physiological state [12, 13].

In cancer research, the assessments of NP-based therapeutics in 2D models for evaluating cellular process may not accurately reflect biological barriers and NPs’ toxicity, which can be overestimated. For instance, Chia et al*.* investigated ZnO NPs in both 2D and 3D models of colorectal cancer and found that NPs in the 2D models exhibited higher toxicity compared to 3D models [14]. According to the results, 3D models more accurately demonstrated the cell-extracellular matrix (ECM) interactions, which likely played a critical role in toxicity determination. Similarly, assessments of the effective delivery of NPs between 3D tumor spheroids and in vivo models also demonstrated important similarities between the two techniques. For examples, lipid NPs encapsulating the photosensitizer verteporfin significantly reduced the viability of ovarian tumor spheroids and inhibited tumor cell proliferation in an animal model of ovarian cancer compared to the free drug [15]. Hepatocellular tumor cells in 3D hydrogels showed higher resistance to treatment with biotin-conjugated pullulan acetate nanoparticles (Bio-PA NPs) compared to the 2D models [16]. Moreover, these NPs showed similar antitumor effects in 3D cultures and xenografted animal models [17]. Overall, these findings underscore the potential of 3D models for assessing nanomedicine efficacy.

**Fig. 1:**
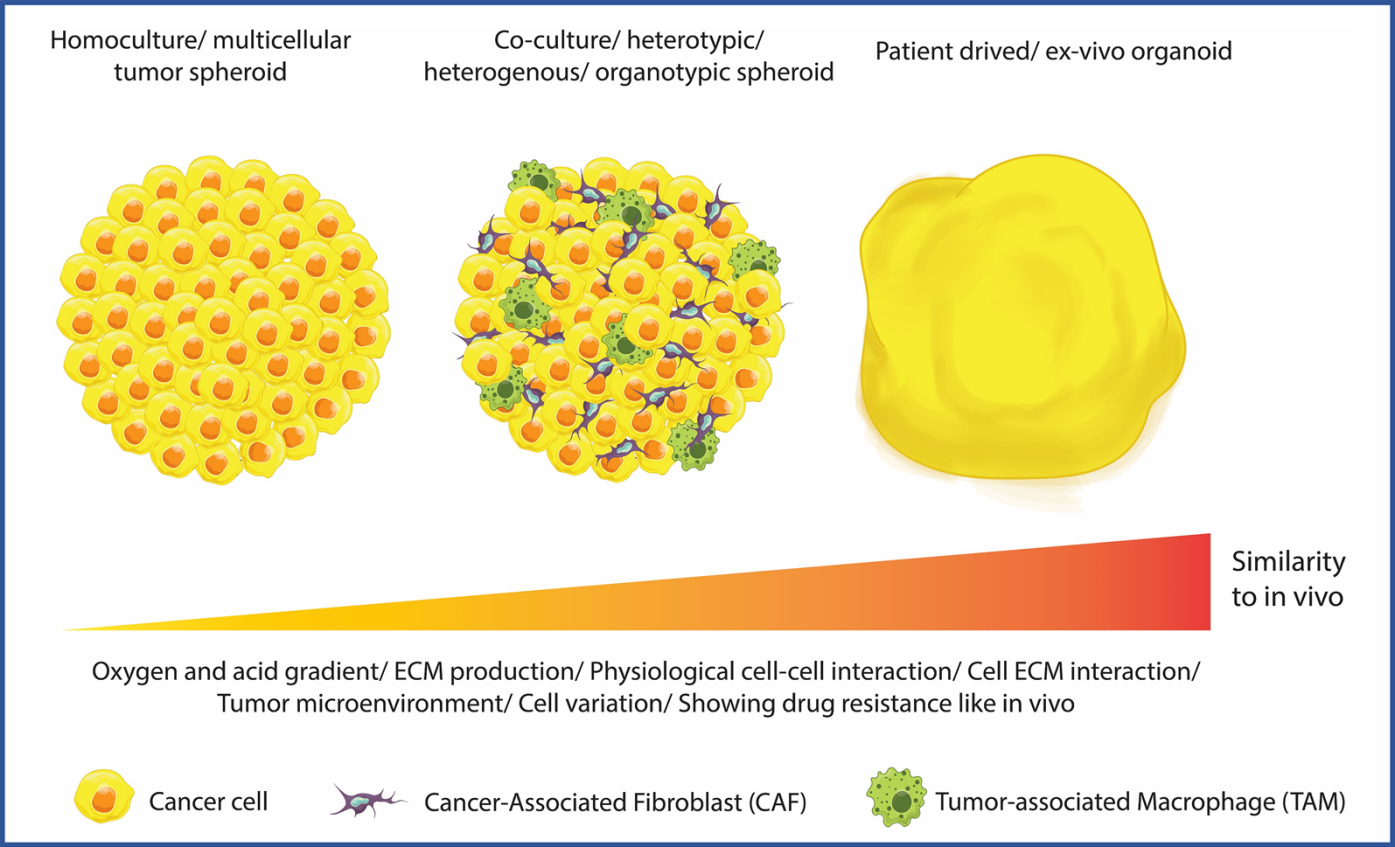
Spheroid tumor models can have different levels of complexity

Most of the studies presented in the literature have utilized spheroids consisting of only one tumor cell type. However, due to the complex nature of tumors with various cellular and non-cellular elements, monoculture spheroids are unable to replicate the complexity of tumors. Consequently, 3D tumor spheroid models with different levels of complexity ranging from monoculture spheroids to heterotypic (co-culture) spheroids and patient-derived ex vivo organoids have been developed (Fig. 1) [17, 18]. Hence, the use of a heterotypic platform for nanomedicine assessment that integrates various types of cells representing the vasculature (e.g., endothelial cells), the immune system (e.g., macrophages), and ECM production (e.g., fibroblasts) is strongly recommended [19]. This review provides an overview of the different 3D co-culture spheroid models proposed in the literature and discusses their utilization in the field of nanomedicine.

## What features are simulated by heterotypic spheroids which make it a suitable model for nanomedicine?

The healthcare industry faces numerous challenges, including drug resistance, side effects from existing therapies, and increased medication costs. As a result, the need to develop new drugs has become more pressing. The use of nanoparticles in medical research has revolutionized treatment approaches. By combining nanomedicine with knowledge from structure-based drug design more effective and precise treatment plans are expected to emerge [5]. In cancer research, several in vitro methods are employed to evaluate the effectiveness of nanomedicines, including assessing cytotoxicity, invasion inhibition, and cell uptake capacity based on drug mechanisms and cell models [20]. However, most current in vitro research uses 2D tumor cells, making it difficult to predict how tumor cells will respond to therapy. To address these limitations, an efficient model for evaluating nanomedicine performance is required, and a comprehensive understanding of the harmful effects of NPs must be obtained. 3D models can help overcome these challenges, as they can be used to analyze medication resistance, assess tumor development, and determine the spread of tumor metastases.

The behavior of NPs in 3D models can vary depending on the nature of the NPs, their physicochemical properties and the ligands bound to their surface. Additionally, tumors exhibit various anticancer drug resistance mechanisms including metabolic drug inactivation, downregulation of specific therapeutic tumor targets, overexpression of cell membrane efflux pumps, and increased activation of DNA repair pathways [21]. Furthermore, the abnormal tumor vasculature, resulting in interstitial hypertension in the core of solid tumors, and the thick ECM protect cancer cells from cytotoxic compounds by reducing drug penetration depth. Other inherent obstacles include the tumor's microenvironment, which is acidic and has a low O_2_ pressure, intrinsic cellular diversity, and interactions between cancerous cells and the stroma, which enhance tumor microenvironment (TME)-mediated drug resistance and restrict the therapeutic effectiveness of both chemotherapeutic medicines and nanodrug delivery systems.

To evaluate the therapeutic efficacy of a particular nanodrug, it is essential to assess critical parameters, such as the targeting capability and the rate of drug penetration due to the wide diversity of nanomedicines and the complexity of tumor tissue [22]. To determine which in vitro model is most appropriate, what criteria must be considered, and what analysis must be conducted, it is important to examine NPs for their penetration, cytotoxicity, and other relevant parameters in vitro [22].

**Fig. 2:**
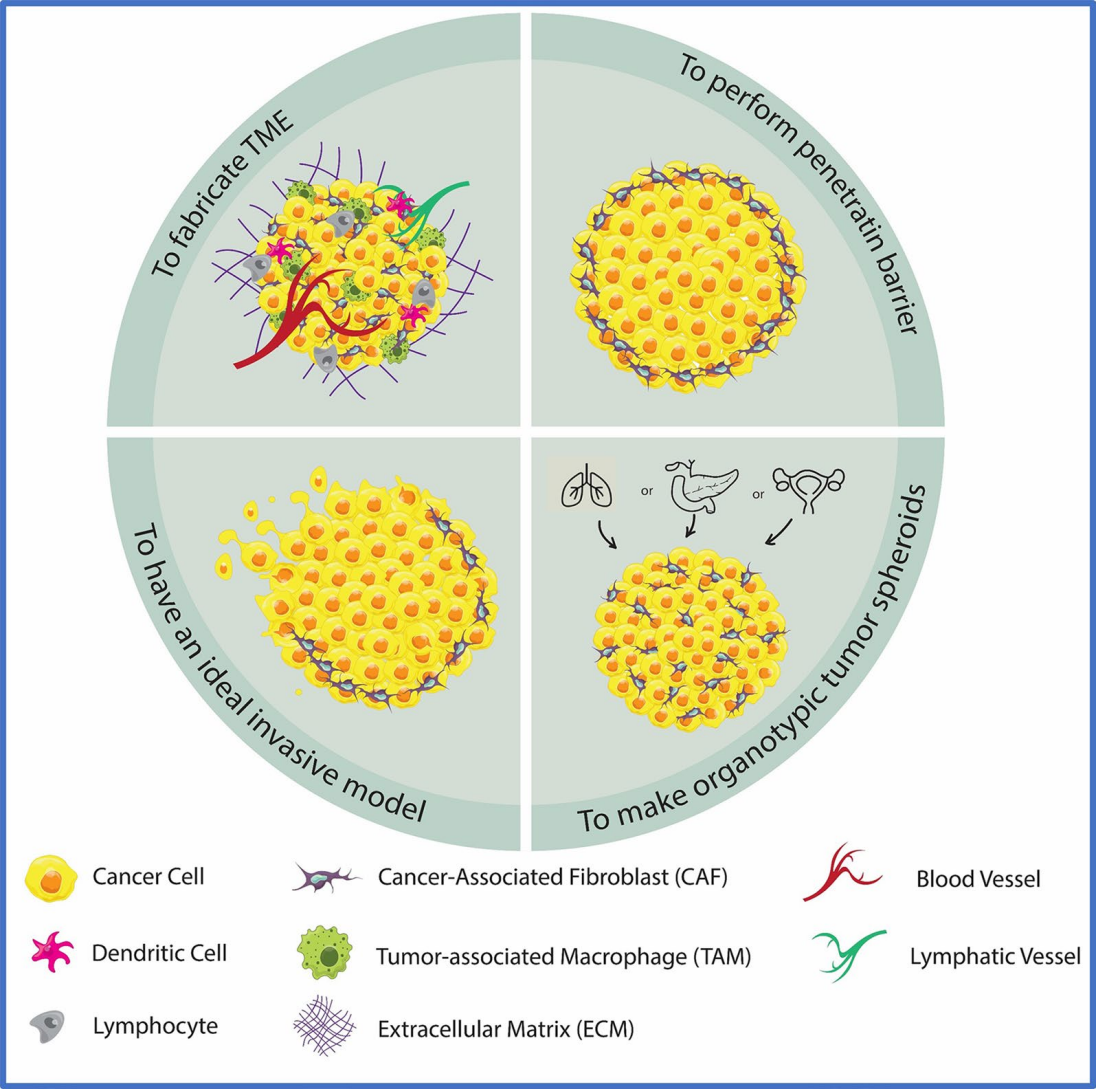
Different applications of applying co-culture spheroids in nanomedicine studies

Since parameters such as cell type, number of cells, presence of scaffold, and nearly cell arrangement can be controlled during the production of co-cultured spheroid, this cell model has been used to evaluate the efficacy of nanomedicines for diverse proposes. In the research articles, four main goals, including modeling the microenvironment, controlling penetration, modeling various organotypic spheroid models, and producing spheroids to explore the invasive behavior of cancer tumors, have been investigated. These four goals are described below and briefly depicted in Fig. 2**.**

### Modeling the tumor microenvironment

TME is a rather complex and heterogeneous ecosystem that comprises not only malignant cells but also a set of cellular and non-cellular elements that dynamically interact with the cancerous cells and can elevate tumor growth and metastasis [23].The TME that regulates tumor development and invasion and is made up of a cellular component consisting of normal fibroblasts and cancer-associated fibroblasts (CAFs), endothelial cells from the lymphatic and blood arteries, and both adaptive and innate infiltrated immune cells. The non-cellular part includes signaling molecules and ECM components. The ECM is a three-dimensional array of glycoproteins, elastin, collagen, proteoglycans, and glycosaminoglycans that provide structural and biochemical support to surrounding cells and is remodeled by the growing tumor [21]. The interaction between cancer and stroma is influenced by both chemical parameters (various cytokines, growth and angiogenesis factors, and molecules involved in cellular adhesion) and physical parameters (structural stiffness, cell contraction and expansion, and cell topology).

In recent years, the TME has been considered in the development of effective medicines for treating advanced tumors due to its role in cancer progression, resistance to therapy, and influence on the enhanced permeability and retention (EPR) effect [21, 24]. Certain characteristics and components of the TME can be employed to better directing nanomedicines to the tumor location. ECM components, such as different glycoproteins and metalloproteinases, as well as tumor-infiltrating immune cells, including tumor-associated macrophages (TAMs) and antigen-presenting cells (APCs), are attractive therapeutic targets. An alternative strategy to enhance the selectivity of drug delivery involves the development of stimuli-responsive NPs, which take the advantage of the general properties of the TME's. Therefore, trigger-dependent nanocarriers that initiate drug release only when exposed to specific properties of the TME, such as an acidic and abnormal redox microenvironment or activated metalloproteinases, can be used to control drug release in a highly precise manner [25, 26].

Before being approved for human trials, potential anticancer nanomedicines must be investigated in preclinical models simulating the TME and mouse models containing tumor xenografts [26]. Therefore, a reliable in vitro model of cancer should include not only cancerous cells but also a variety of stromal cells and ECM components. The spheroid monoculture approach is not able to perfectly recreate the conditions present in tumor cells. As a result, various intricate spheroids-based models have been developed to imitate the cellular heterogeneity found in tumor tissues [27].

**Fig. 3:**
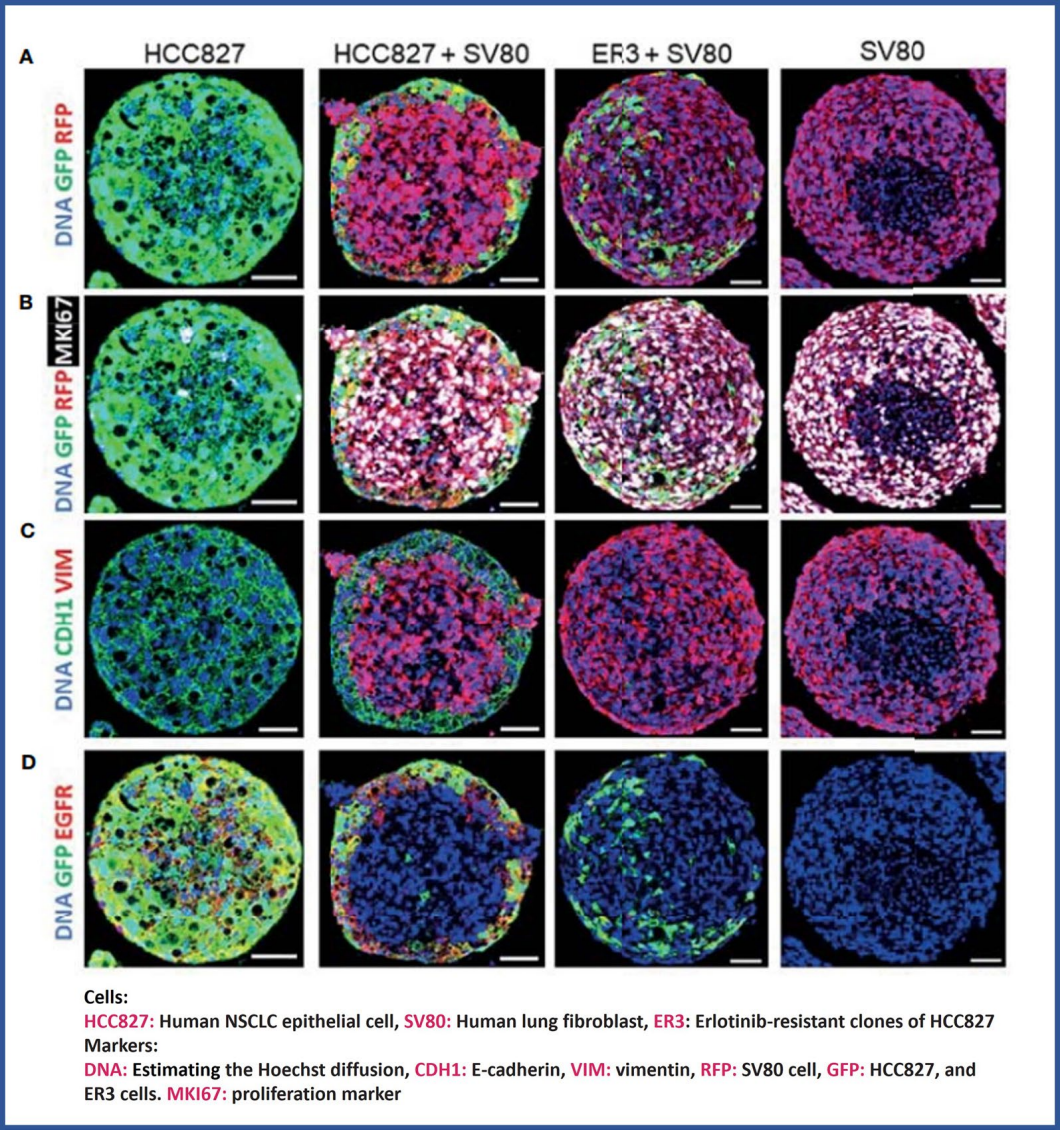
The immunohistochemical and mass cytometry imaging techniques were utilized to characterize both homotypic and heterotypic spheroids. **A** Upon coculturing with fibroblasts, ER3 and HCC827 cells were distributed sparsely and localized towards the periphery, respectively. **B**: The expression of MKI67 marker, colocalized with GFP and RFP, revealed an elevated proliferation rate of SV80 fibroblasts. **C** Vimentin expression was exclusively observed in the mesenchymal fibroblasts while E-cadherin expression was limited to HCC827 cells. **D** The expression of EGFR was detected in both mono- and co-culture spheroids containing HCC827 or ER3 cells, but not in SV80 monoculture spheroids, reproduced from Ref [31], under the terms of the CC B

Several review papers have been published that emphasize the importance of the TME in spheroid and other 3D cell culture models [28–30]. However, due to the complex nature of the TME, the vast number of cells involved, and the varied mechanisms of drug delivery, further studies are needed to fully characterize the role of different TME components in drug delivery and evaluate the efficacy of novel nanomedicines. To address this issue, Lotsberg et al*.* recently conducted an interesting research study in which they produced co-culture spheroids to replicate the tumor TME model [31]. The research focused on exploring the mechanisms of EGFR inhibitor resistance and the impact of cancer stroma interactions between fibroblasts and drug-sensitive or drug-resistant cancer cells, which are not yet well understood. The team created and characterized 3D homotypic and heterotypic spheroid models using EGFR inhibitor (EGFRi)-sensitive or EGFRi-resistant non-small cell lung cancer (NSCLC) cells. The researchers utilized the HCC827 cell line, its various clones, and two human lung fibroblast cells to generate homotypic and heterotypic spheroids. They employed immunohistochemistry and imaging mass cytometry methods to characterize the multicellular heterotypic spheroids comprehensively. The findings of this research revealed inherent differences between epithelial and mesenchymal-like cancer cells when co-cultured with fibroblasts, specifically related to how they sort themselves, organize over time, and interact with stromal cells. Furthermore, the researchers observed that the development of mesenchymal features as a resistance mechanism against EGFR inhibitors is inversely correlated with the cells' ability to form compact multicellular spheroids in this model system (Fig. 3).

### Modeling the penetration barrier

The absence of tumor-like penetration barriers in 2D cell culture is a limitation that leads to misleading drug sensitivity and an inability to predict therapeutic potential. Most solid tumors have high tumor interstitial fluid pressure (TIFP) due to irregular vascularization, ECM stiffness, and limited lymphatic drainage. This pressure increases drug clearance from the tumor extracellular space, resulting in low drug accumulation in deep tumor nests. The low therapeutic efficacy of NPs may be attributed to the difficulties of drug delivery methods in crossing physiological barriers and reaching hypoxic and necrotic zones of tumors located far from blood vasculature, [32]. Furthermore, nanocarriers' physical features, such as size, shape, charge, and chemical composition, influence their penetration, and examining each parameter separately and in combination is important.

Although drug permeability experiments in animal models for accumulation evaluation may be advantageous, their high cost and time-consuming techniques make them challenging to apply. Additionally, the drug carriers' pharmacokinetics and therapeutic efficacy may be overestimated due to the leakier vasculature of mouse tumors compared to human tumors. Therefore, producing in vitro models to investigate the penetration of NPs into tumor tissue is of great interest and importance. Co-culture spheroids hold promise for drug permeability studies, with the ability to alter the ratio of cells affecting nanodrug permeability. Fluorescent imaging technologies, such as confocal and light sheet microscopy, are practical approaches for evaluating nanomedicine penetration. The ability of nanocarriers to penetrate and accumulate in various tumor’s cell layers to produce a long-lasting impact is critical for current cancer-targeting therapeutics [33].

**Fig. 4:**
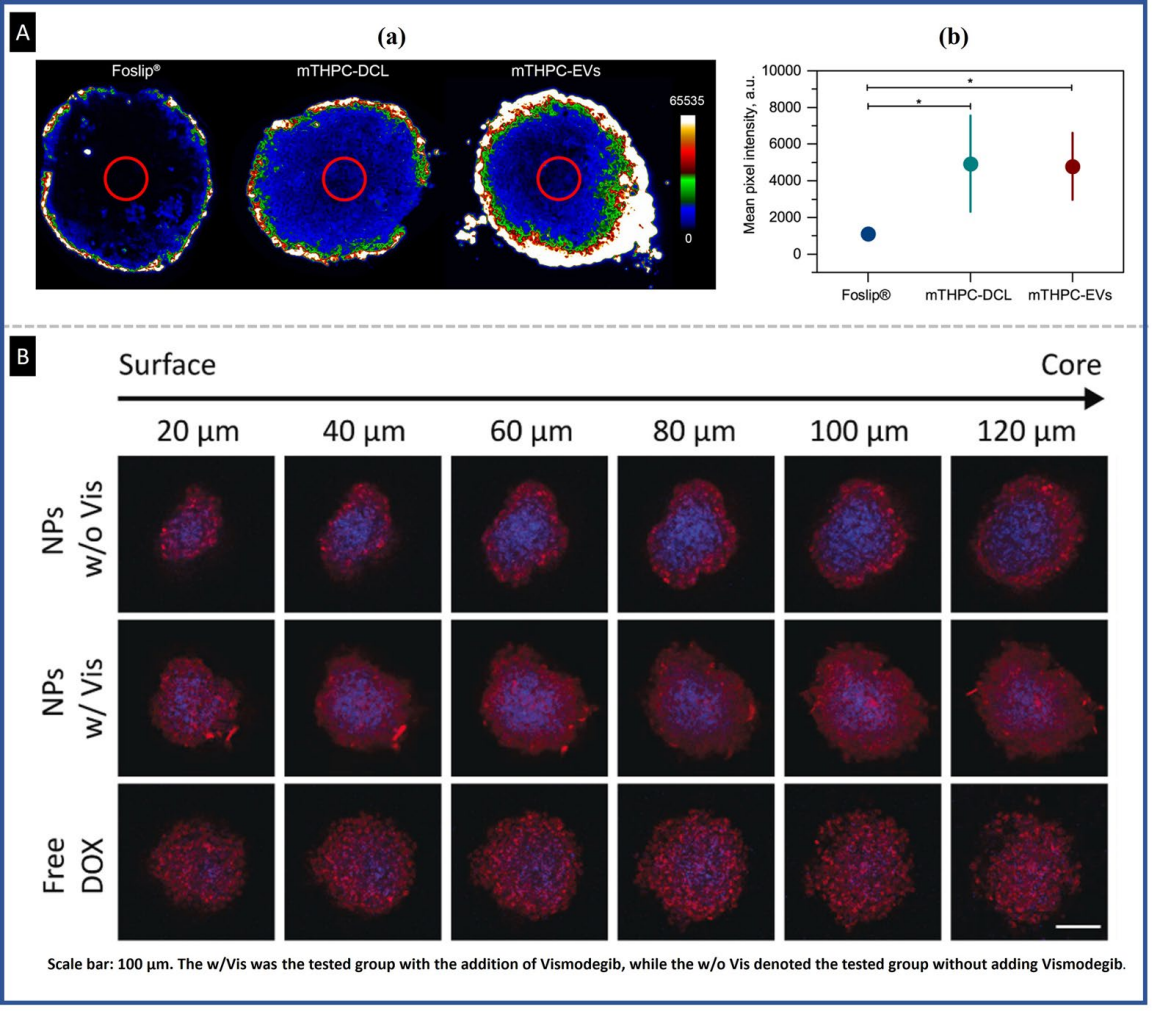
Heterotypic spheroids are a suitable model for studying the penetration of drugs/nanodrugs: **A** Comparison of three nanoliposomes' penetration in Fadu:Mewo spheroids in 3 days after seeding and 24 h after drug incubation. (a) Fluorescence pictures of cryosections of Fadu:Mewo spheroids; (b) Quantification of temoporfin fluorescence intensity in spheroids' cores (red circles), reproduced from Ref. [35], under the terms of the CC BY. **B** The confocal microscopy-based captured the penetration of nanoparticles in co-cultured spheroids, with the red and blue channels representing DOX and nucleus, respectively. Notably, in the group treated with DDS encapsulated with Vismodegib and DOX, an even distribution of red signal was observed in the tumor spheroid core, indicating effective delivery of the drug [36],, Copyright 2016 Elsevier.

N. Ho et al. produced an endothelial based core–shell spheroid to test the permeability of tumstatin-Fe_3_O_4_ NPs. After developing the RG2 3D spheroid as a core, they placed a layer of BPAE endothelial cells on these cells via self-assembly associated with intercellular interactions [34]. They found that these NPs had selective targeting capacity for the endothelial cell layer and greater neo-vascularization inhibitory effectiveness than free tumstatin. Yakavets et al*.* evaluated the permeability of three different liposome-based carriers of temoporfin in co-cultured spheroids [35] and found that the penetration of NPs in spheroids varied based on the carrier used (i.e., 49.7, 87.8, and 47.8 µm for extracellular vesicles, cyclodextrin-based liposomes (mTHPC-DCL), and Foslip (mTHPC-EVs), respectively). They also evaluated the amount of drug delivery and obtained important parameters for improved drug optimization by examining the kinetics and depth of penetration of NPs as a function of stromal content. Figure 4A compares the penetration depth of the three types of nanoliposomes in the spheroids. The ability of nanocarriers to penetrate and accumulate in various tumor cell layers to produce a long-lasting impact is critical for current cancer-targeting therapeutics.

Hsieh et al*.* evaluated the multi-responsive nanodrug using spheroids prepared through co-culturing MDA-MB-231 breast cancer cells and DFs dermal fibroblast cells in a ratio of 3:1 [36]. They developed a drug delivery system (DDS) using a polypeptide nanoparticle that is responsive to endogenous stimuli. Due to the upregulated expression of certain enzymes and reductive agents, the nanoparticle is designed to dissociate in the tumor microenvironment. The DDS comprises two polymeric sequences that self-assemble into nanoparticles in an aqueous phase, and the authors used this multi-functional delivery system to encapsulate Vismodegib and DOX. After the cancer cells take up the multi-responsive nanodrug, the nanoparticles are dissociated by overexpressed GSH in the lysosome, releasing Vismodegib. This drug then binds to the SMO receptor and down-regulates the hedgehog signaling pathway, which can significantly improve stromal dissociation in solid tumors. To examine how nanoparticles were distributed in heterotypic spheroids of varying diameters, a confocal microscope was utilized to observe the depth of drug penetration. As depicted in Fig. 4B, the penetration ability varied depending on the presence of Vismodegib. After six hours of incubation, red fluorescence from DOX was detected at the center of the spheroid in the group treated with Vismodegib-encapsulated nanoparticles. In contrast, without Vismodegib, the nanoparticles remained at the tumor spheroid's periphery and could not penetrate as deeply. This suggested that Vismodegib alleviated tumor stroma and fostered a favorable microenvironment for nanoparticle transportation (Fig. 4B**)**.

### Modeling the cell invasion

Tumor invasion is a critical phase in tumor metastasis since cancerous cells must leave the primary tumor to invade their surroundings or enter circulatory systems [37]. Extensive research has shown that the invasive potential of tumor cells is correlated with their cellular/nuclear morphology and TME. Numerous methods, including scratch assay, transwell invasion assay, colony assay, and spreading assay have been developed to better understand the involvement of the TME in tumor invasion. Through these assays, we gained a lot of evidence on the key factors that drove and control tumor invasion inside the TME. However, the impact of tissue architecture in tumor invasion is poorly understood.

**Fig. 5:**
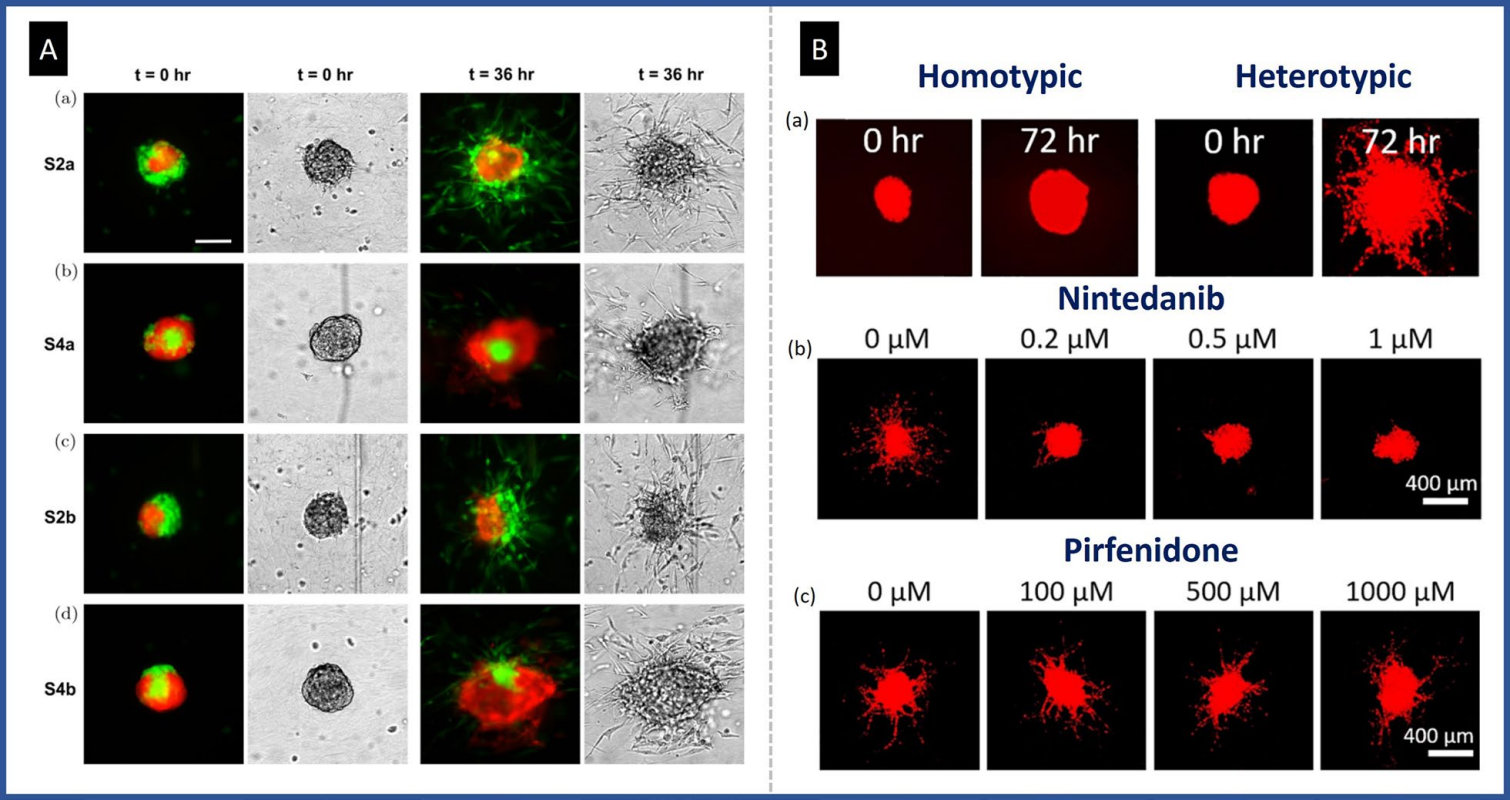
Utilizing the co-culture model for the purpose of investigating the invasive behavior of cancer cells. **A** Study how the architecture of co-cultured spheroids (MDA-MB-231, shown in green, and MCF-10A, shown in red) affects tumor cell invasion in collagen matrices. **a** Significant numbers of MDA-MB-231 cells invaded the spheroids with metastatic cells outside a non-cancerous cluster. **b** Only a few peripheral MDA-MB-231 cells invaded out of the spheroids with reverse architecture. **c** If two cells are uniformly placed on the two sides of the spheroid, the invasion occurs from the side containing the metastatic cells. **d** Metastatic cells act invasively from any exposed area if the covering of non-cancerous cells is incomplete, reproduced from Ref [32], under the terms of the CC BY. **B** Investigation the role of fibroblast in establishing invasion and targeted therapy using confocal fluorescent microscopy. cancer cells only were labeled with live red fluorescent dye, panel (**a**) shows A549 spheroids in monoculture and co-cultured with fibroblast in fibrin/Matrigel matrix. (**b**) Illustrates the effects of various concentrations of cisplatin with and without 0.5 µM Nintedanib on co-culture spheroids within fibrin/Matrigel. reproduced from Ref [38], under the terms of the CC BY

Due to better modeling of the TME, the co-culture spheroid has an advantage over conventional models for examining the architectural factor of cells' invasive behavior. For example, Huang et al*.* created remarkable 3D co-culture models that mimicked physiologically relevant TME conditions, for performing an invasion test. They created a co-culture spheroid with a 1:1 ratio of triple-negative breast cancer MDA-MB-231 cells, and non-cancerous epithelial MCF-10A cells, and subsequently placed it in a collagen matrix in a microfluidic device [32].The spatial distribution of the two cell types in co-culture spheroid was found to interestingly control the tumor invasion, which demonstrated by real-time imaging over a time course of 36 h. In their experiment, four different patterns of cell architecture were obtained in co-culture spheroids, which were significantly different in invasion ability (Fig. 5Aa–d). The result showed that cell invasion in a collagen matrix was affected by the shape of co-culture spheroids. When the non-cancerous MCF-10A cells cover the metastatic core, the metastatic MDA-MB-231 cells are restricted and prevented from migration.

In another recent study conducted by Pan et al., the heterotypic spheroid model was employed to evaluate the effectiveness of therapeutics targeting the TME in the context of cancer treatment [38]. Specifically, the study aimed to assess the impact of two anti-fibrotic drugs, Nintedanib and Pirfenidone, on the growth and invasion of lung cancer cells co-cultured with fibroblasts. The study employed a heterotypic spheroid model consisting of fibroblasts and lung cancer cells in Matrigel supplemented with fibrin to achieve this. The authors quantified spheroid irregularity using the inverse of spheroid circularity (1/C value). They observed that co-culture with fibroblasts significantly increased the average 1/C value to 31.5 after 72 h, compared to homotypic spheroids with an average 1/C value of 3.2. This suggests increased invasiveness of cancer cells when interacting with fibroblasts (Fig. 5Ba). According to their analysis of the effects of anti-fibrotic agents in conjunction with cisplatin, only Nintedanib was found to effectively suppress the growth of cancer cell spheroids and inhibit cell invasion (Fig. 5Bb). Moreover, Nintedanib demonstrated superior performance compared to Pirfenidone by significantly reducing the expression of four genes within fibroblasts that are known to be involved in critical cellular functions such as cell adhesion, invasion, and extracellular matrix degradation. These genes are collagen V, collagen I, fibronectin, and FKBP10.

### Modeling 3D organotypic cancer tissue

Organotypic cancer tissue models are composed of two or more cell types, often tissue-derived cells, along with matrix-like elements to simulate the extensive interactions that occur in the tissue [39]. To more accurately mimic the architecture of solid tumors in terms of structural and biochemical TME features, 3D organotypic spheroid models can be built from primary cells. The cell sources used to create organotypic spheroids vary, from cell lines that can be activated using different signaling molecules to primary cells and patient-tissue-derived cells. Singh et al*.* developed an organotypic spheroid model that replicates the TME of breast tumors by co-culturing human primary activated breast fibroblasts, breast cancer cells, and collagen [40]. To determine the optimal cell/matrix composition for developing realistic organotypic breast cancer spheroids, they investigated the effect of fibroblast activation, fibroblast cell types, stromal ratio to cancer cells, and collagen concentrations. Furthermore, they discovered that oncogenic MAPK pathway-driven ECM invasion by cancer cells requires both CXCR4 and CCXCL12 in the TME and that suppressing tumor-stromal signaling with a specifically targeted drug may be used as a therapeutic method to reduce breast cancer cell invasion [40].

**Fig. 6:**
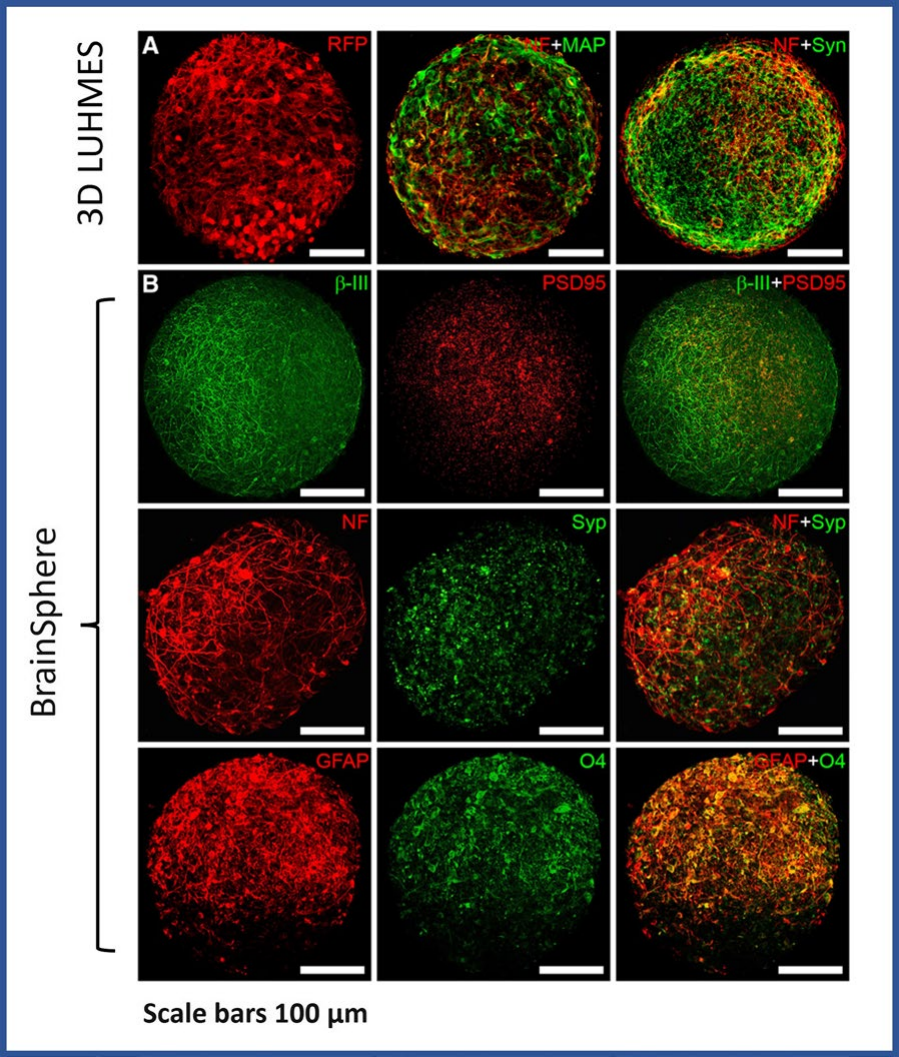
The confocal images of two brain spheroids: (**A**) 3D LUHMES cell after 7 days of differentiation, which is expressing RFP or has been stained with various neuronal markers such as MAP2 (in green), neurofilament (in red), and synaptophysin (in green). (**B**) the BrainSpheres model after 4 weeks of differentiation, which exhibits a range of neural cell type markers, including β-III-tubulin/PSD95, neurofilament/synaptophysin, and GFAP/O4, reproduced from Ref [41].under the terms of the CC BY

In their quest to develop accurate and clinically relevant models for assessing the penetration of different nanoparticles into neural cells, Leite et.al., prepared two novel 3D human brain spheroids [41]. The first model featured human dopaminergic neurons that were differentiated from the LUHMES cell line, and it was named 3D LUHMES. The second model, known as BrainSpheres, was derived from human induced pluripotent stem cells (iPSCs) and represented a more sophisticated and realistic neural structure. The 3D LUHMES model exhibited a neuronal morphology with multiple cellular projections and expressed markers of matured nerve cell, such as neurofilament, MAP2, and synaptophysin, after just 7 days of differentiation. After 4 weeks of differentiation, the BrainSpheres model demonstrated a diverse range of neural markers consistent with various neural cell types, including neurons, astrocytes, and oligodendrocytes (Fig. 6).

## Fabrication

Various methods for producing co-culture spheroids have been introduced with direct mixing and core–shell approaches being commonly used for drug evaluation. In the direct mixing approach, cancerous cells are mixed with other cells in a desired ratios and placed in conditions suitable for spheroid formation, such as ultra-low attachment (ULA) plates or hanging drop plates, with multiple cell types and/or scaffolds potentially included. Encapsulating cell mixtures in droplets of defined size is another approach for producing mixed co-culture spheroids. Droplet-based microfluidic devices are particularly appealing for this purpose, as they offer control over droplet diameter and content and can facilitate cost-effective and high-throughput screening. For instance, Lee et al. utilized a tree-shaped gradient generator to regulate cell density and encapsulate different cells in droplets of uniform size [42]. This device enables the efficient production of uniform-sized 3D tumor spheroids with varying cellular ratios, which can be used to assess the cytotoxicity of anti-cancer drugs.

**Fig. 7:**
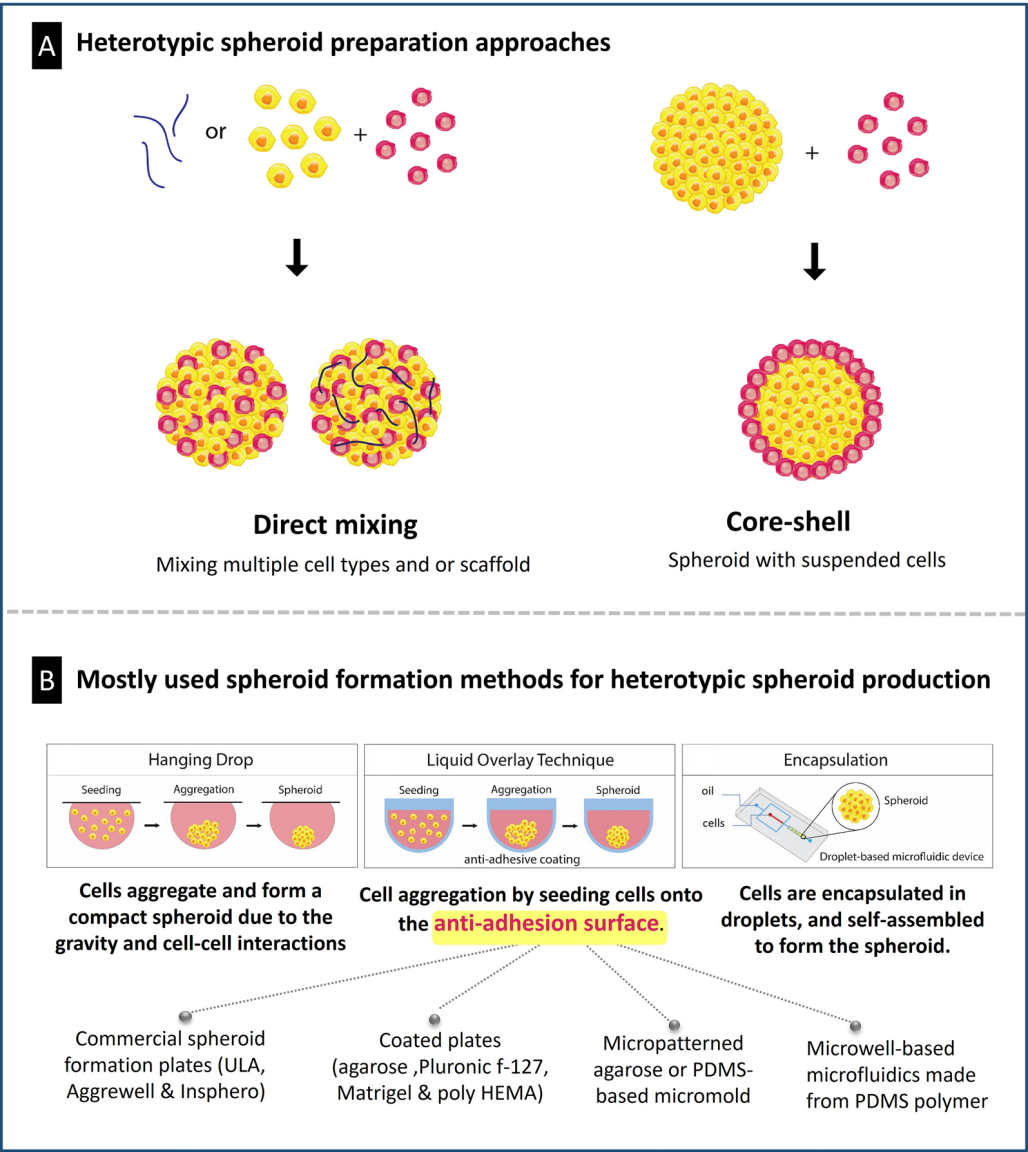
Co-culture spheroid production methods. **A** Types of co-culture approaches; **B** types of spheroid production methods that are commonly used in co-culture spheroid production processes

In core–shell approach, pre-formed tumor spheroids are placed on top of a monolayer of other cells or entered a suspension of other cells to generate a core–shell structure (Fig. 7A). Hanging drop and liquid overlay techniques have been extensively used for co-culture model development among other spheroid fabrication methods (Fig. 7B) [36, 37]. The hanging drop method is a simple technique for spheroid formation, where flipping a universal 96-well plate can easily prepare spheroids without require any special instruments. This method can incorporate any biological or chemical agent that may impact intercellular or cell-ECM connections can be used to prepare co-culture of various cell types to investigate the significance of cellular/matrix contacts in determining spatial relationships between cells [43].

The liquid overlay method involves creating a non-sticky surface to avoid cellular attachment, allowing cells to bind to each other and form aggregates that eventually become spheroids [44]. Non-adhesive surfaces can be obtained using commercially available low attachment cell culture plates, such as ULA 96-well microplates, AggreWell plates, and/or InSphero microplates. Coating plate surfaces with hydrophobic polymers, such as agarose, pluronic F-127, matrigel, and poly HEMA, can also achieve non-adhesive surfaces and spontaneous spheroid formation of uniform size. Micropatterned agarose scaffolds can also be fabricated using a micromolding method to obtain a non-adhesive surface for spheroid formation, enabling precise control over size, density, and mass of individual spheroids [45]. Finally, microfluidic devices made from polydimethylsiloxane (PDMS) can generate large-scale homogenous spheroids in a uniform manner [46–49]. PDMS is widely used due to its superior optical transparency, manufacture capability, high gas permeability, and biocompatibility, making it a common material for constructing microfluidic chips for cell culture [50].

## Application of tumor co-culture spheroid models in nanomedicine

### Cross talks between cancer cells and their neighbors

Generating a cancer model requires careful consideration of how cancer cells behave when grown in co-culture. The TME is characterized by heterogeneity in terms of both cellular and non-cellular components, which means that tumor cells alone cannot account for cancer initiation, progression, metastasis, and drug resistance [51]. For instance, co-culturing human pancreatic stellate cells (hPSC) with a pancreatic cancer cell (Panc-1) resulted in an increase in the expression of KI-67, fibronectin, and smooth muscle actin (a-SMA). The results suggested that the interactions between hPSC and Panc-1 led to an increase in proliferation and activation of hPSC highlighting the potential of co-culture cultivation for reforming the TME [52]. Similarly, Gao et al*.* demonstrated that cancer-associated fibroblasts (CAFs) promoted early peritoneal metastasis of ovarian cancer in a heterotypic culture model [53].

In the TME, various non-tumor stromal cells continuously interact with tumor cells, including CAFs, immune, and endothelial cells. Incorporating these non-tumor cells in 3D models would improve the accuracy of cancer models, as different cells may react differently to treatment due to reciprocal interactions with neighboring cells in TME [54].

#### Fibroblast-cancer cell interaction

**Fig. 8:**
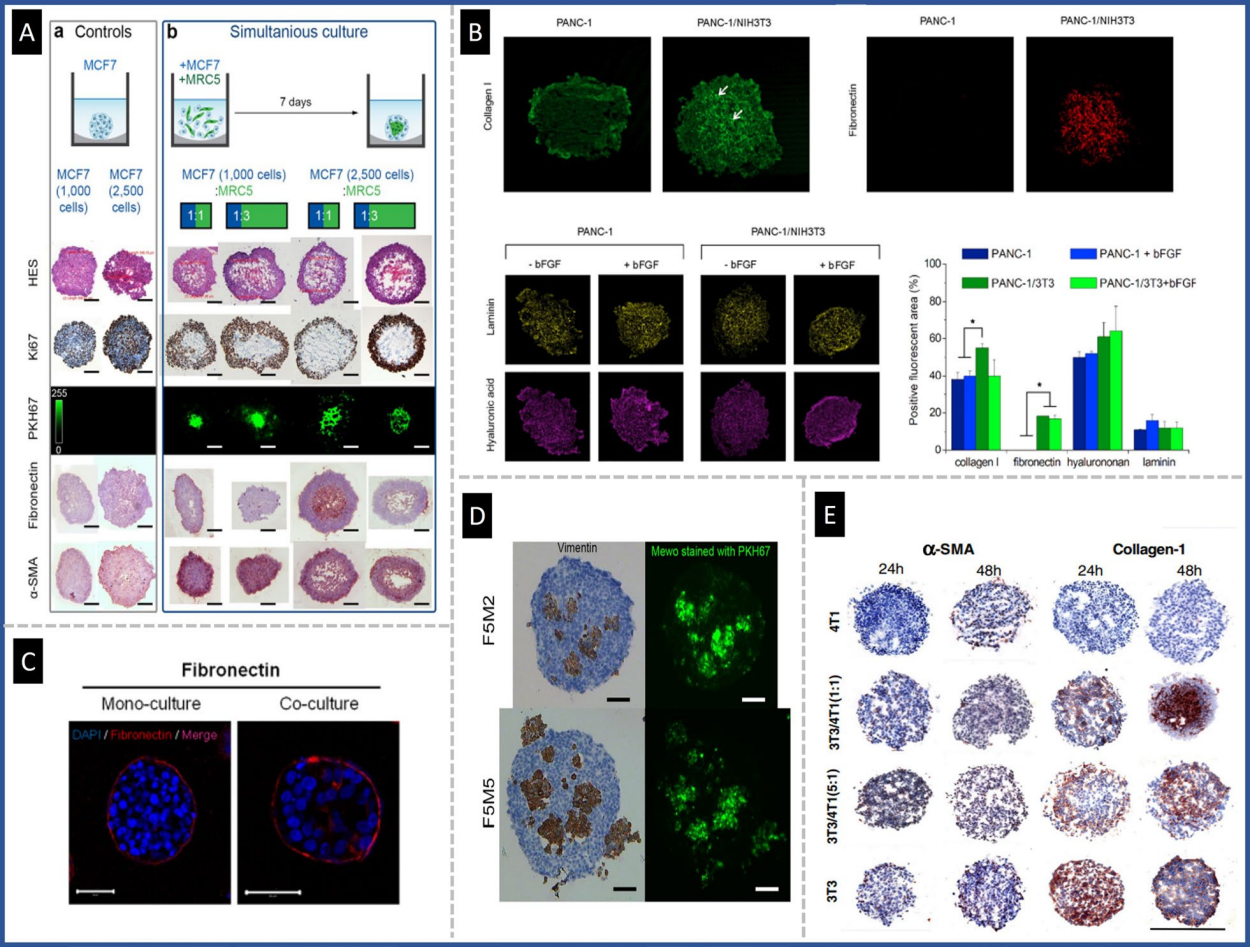
Inclusion of fibroblasts in 3D spheroid co-culture models of cancer and the expression of stromal biomarkers. **A** Characterization of MCF7 monocultures (**a**) vs. MCF7 and fibroblast cells (MCR5) co-culture model (**b**), reproduced from Ref. [57], under the terms of the CC BY. **B** Expression of ECM components of collagen-1, fibronectin hyaluronan, and laminin in pancreatic cancer spheroids (PANC-1 and PANC-1/NIH3T3 spheroids), Reproduced with permission from Ref [58], Copyright 2019 Elsevier. **C** Consequence of co-culturing fibroblast on the expression of fibronectin, reproduced from Ref. [59], under the terms of the CC BY. **D** Effect of fibroblast co-culture on the expression of vimentin marker, reproduced from Ref. [35], under the terms of the CC BY. **E** Images for the immunostaining of both α-SMA and collagen-1 in homo- and heterotypic spheroids for evaluation of stromal marker, Reproduced with permission from Ref [60], Copyright 2016 Elsevier

Among the various cellular components that constitute the TME [55], CAFs have emerged as a key player in cancer progression, prompting numerous studies investigating stromal-tumor cell interactions and their incorporation into in vitro models. CAFs primarily participate in ECM deposition and degradation within tumor tissue, making them key players in tumor invasion and resistance [56]. Figure 8 depicts the impact of including fibroblasts in co-culture models and the expression of stromal biomarkers in 3D spheroids of different cancers.

**Fig. 9:**
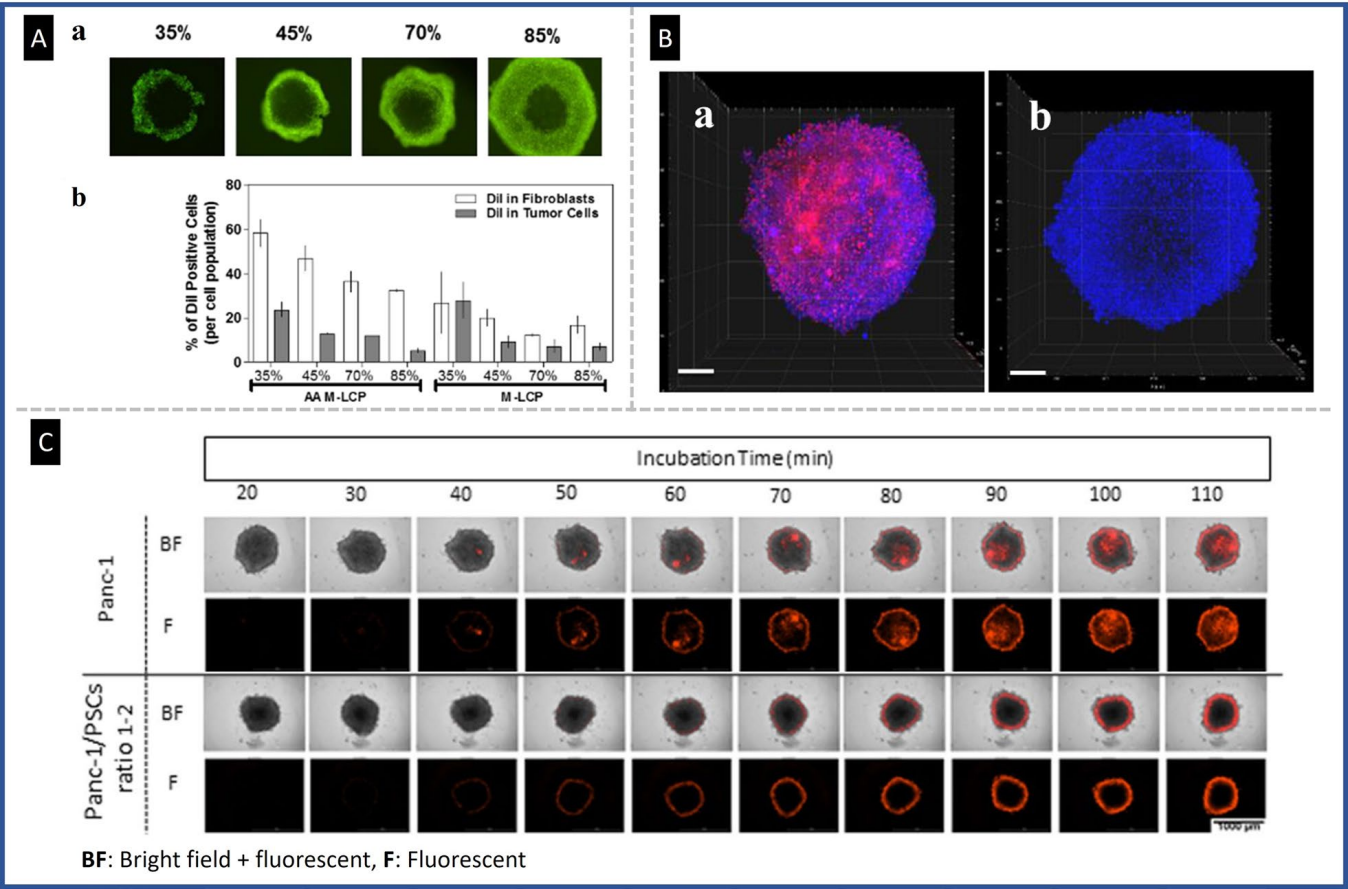
The relationship between the presence of fibroblasts and penetration of nanodrugs in co-culture spheroids. **A** Changes in spheroid morphology as fibroblast densities increased (**a**); and the correlation between DiI-labeled AA-LCP NPs and spheroids containing varying numbers of fibroblasts (**b**). When the fibroblasts ratio was higher, NPs were more interact with fibroblasts, Reproduced with permission from Ref [66], Copyright 2016 American Chemical Society. **B** 3D tomography with LSFM of tri-cultures spheroid after incubation with Dox (**a**) and Dox-loaded NPs (**b**) for 4 h at 37 °C, blue (nuclei, Hoechst) and red (Dox), Scale bars: 200 µm, Reproduced with permission from Ref [70], Copyright 2019 Elsevier. **C** The fluorescence of entire spheroids was followed over time and recorded kinetically after incubation with fluorescently tagged AuNR. For Panc-1, fluorescence rose steadily over all spheroids over time, but in the Panc-1/PSC spheroid (1:2 cell ratio), it lagged and was mostly confined to the outer layers of the spheroids, reproduced from Ref. [78], under the terms of the CC BY

The inclusion of cancer-associated fibroblasts (CAFs) in 3D culture models has prompted numerous investigations into their impact on the migration and invasion of cancer cells. In pancreatic ductal adenocarcinoma (PDAC), stromal components, including CAFs, immune cells, and ECM are known to enhance the aggressiveness of the disease. Lee et al. developed a 3D heterotypic spheroid model of pancreatic tumors with PSCs, the primary source of CAFs in PDAC, within collagen-supported microchannels. It was observed that under co-culture conditions with ECM alternation, tumor spheroids acquired a migratory phenotype accompanied by drug resistance. Therefore, this proposed co-culture model of pancreatic adenocarcinoma may serve as a valuable tool for evaluating migration, drug resistance, and the underlying molecular mechanisms [61]. Similar models have been developed by adding CAFs in co-culture conditions to more accurately mimic the native TME and better predict in vivo drug response. In pancreatic tumor tissue, CAFs are closely associated with all cancerous structures. To mimic these structural features, Fan et al*.* used a 3D co-culture model in which a layer of NIH-3T3 fibroblast was cultured over a core of BxPC-3 pancreatic cancer cells (BxPC-3@NIH-3T3) [62]. The group proposed pH-sensitive polymeric nanomicelles as a novel method for targeting pancreatic tumors by disrupting the integrity of the membrane in a pH-sensitive manner. It was hypothesized that these NPs could permeabilize the stromal barrier surrounding cancerous tissues, thereby resulting in simultaneous eradication of stromal and cancerous cells. Additionally, animal models of xenografted BxPC-3 pancreatic tumors further confirmed the efficacy of these NPs. In another study, Jain et al*.* designed a heparin sulfate (HS) oligosaccharide-based nanovehicle to target EGFR-overexpressed tumor cells in a co-culture spheroid of MDA-MB-468 breast cancer cells and fibroblast cells, given the importance of fibroblast in ECM remodeling. In this study, a layer of fibroblast cells was generated over the cancer cells. The heparinoid-capped fluorescent AuNPs (AF_555_Au@1) effectively traversed the fibroblast coating and targeted cancer cells, demonstrating the effectiveness of the nanovesicle in targeting cancer cells in TME [63]. Winter et al*.* established a spheroid system comprising epithelial ovarian cancer cells and fibroblasts to assess nanovector delivery [64]. The spheroids were then treated with PEG NPs or cell penetrating peptide (MPG) NPs to evaluate the role of oxygen levels, fibroblast activation, and ECM mimetic (PMX) incorporation. This approach may assist in assessing NPs transport according to the ovarian ascites and metastatic environments and provide a means to estimate nanotherapeutic efficacy. Priwitaningrum et al*.* established a 3D spheroid array through cancer cells co-cultured with fibroblasts to simulate an in vitro model of tumor stroma [60]. Subsequently, silica and PLGA NPs penetration were evaluated in this platform. According to the results, the stroma acted as a barrier for the diffusion of NPs, thereby providing a means to evaluate the characteristics of NPs penetration into tumors. The developed spheroid may be utilized to investigate the interaction of tumors and stroma, antitumor features of nanodrugs, and the diffusion/penetration features of drug-loaded nanomedicines. Granja et al*.* proposed a heterotypic breast cancer spheroid composed of cancer cells and normal fibroblasts to evaluate the penetration capacity of mitoxantrone-loaded solid tumor NPs (SLN-Mito) and its ability to induce antitumor effects [65]. The results indicated that in agreement with other works, the inward layer of the spheroid was affected by SLN-Mito at a lesser amount possibly due to the resistance of spheroid to penetration and greater cell–cell interactions. Miao et al. generated a core–shell spheroid model of tumor cells enclosed by fibroblast to stimulate the in vivo condition and quantified the immediate distribution of NPs into tumor layers after extravasation from blood vessels via lipid-coated calcium phosphate NPs (LCP-NPs). The findings of this study suggested that fibroblasts neighboring to blood vessels in stroma-vessel type tumors participated in the role of CAFs as a binding site for NPs. Distribution of NPs in fibroblasts leads to NPs unavailability for tumor uptake (Fig. 9A) [66].

Pautu et al*.* developed a heterotypic spheroid model of lung cancer using lung cancer A549 cells and fibroblasts (MRC5) to investigate the penetration capacity and efficacy of bare or PEG coated Dox-loaded iron carboxylate metal–organic NPs (nanoMOFs) in a 3D tumor model [67]. Li et al*.* developed 3D heterotypic spheroids composed of NIH-3T3 fibroblasts and tumor cells (4T1) by embedding the co-culturing in Matrigel [68]. The findings showed that liposomes with chimeric cell membrane proteins and membrane-bound elastase (NE-LMP) penetrated deep into the focal point of heterotypic spheroids due to the ability of Neutrophil elastase (NE) to degrade ECM and overcome tumor stromal barriers.

Pant et al. developed co-culture spheroids of lung adenocarcinoma cells in combination with lung normal fibroblast and evaluated the potential of the spheroid model for assessing the toxicity of paclitaxel-loaded Eudragit® RL 100 NPs (ENP) [69]. The impact of paclitaxel-ENPs showed greater sensitivity in 2D cultures compared to 3D cultures, emphasizing the importance of spheroid cultures in examining the efficacy and toxicity of drugs and innovative nanomedicines.

In addition to co-culture models, more complex tri-cultures have been developed for nanomedicine evaluation. Lazzari et al*.* developed a tri-culture pancreatic spheroid made of tumor cells, CAFs, and endothelial cells to reproduce the complexity of the tumor tissue with multiple biological barriers [70]. The penetration of doxorubicin (Dox) and Dox-loaded polymer NPs were then investigated in the developed pancreatic spheroid model. While it was confirmed that Dox was capable of reaching the spheroid core, no Dox-loaded polymer NPs were identified in the spheroids, emphasizing the challenge of delivery of nanomedicine through biological barriers. These results were consistent with previous observations showing that large micelles hardly diffused in fibrosis-rich pancreatic tumors in vivo (Fig. 9B) [71].

Hartwig et al*.* developed a Cu(DDC)_2_ liposomes that exhibit anticancer properties [72]. To assess the cytotoxicity of these liposomes, co-culture neuroblastoma spheroid models composed of neuroblastoma cells in conjunction with fibroblasts and myoblasts were used to simulate TME properties. The results indicate that Cu(DDC)_2_ liposomes effectively increased the toxicity of neuroblastoma spheroids while affecting both neuroblastoma and surrounding normal cells. Dehghankelishadi et al*.* developed a co-cultured spheroid model of head and neck cancer cell line (UM-SCC-1) and NHLF fibroblast on ultra-low attachment surface plates. The group developed high-density lipoprotein NPs (HDL NPs) to deliver miR-34a (miR-34a-HDL NPs) as a radio-sensitizer due to their ability to target pathways associated with radio-resistance. Augmented apoptosis and interrupted cell cycle were observed upon miRNA delivery using these NPs. The improved outcomes observed in spheroids demonstrated the effectiveness of HDL NPs as a promising approach for radio-sensitizing RNA delivery [73]. Rizzo et al*.* developed in vitro monotypic and heterotypic cultures of tumor cells and stromal cells of the pancreas as a model for extracellular pH sensing. The platform demonstrated the feasibility to image multiple types of live cells in a 3D environment and interpret actual pH metabolic interactions under controlled experimental conditions, making it an appropriate model for drug screening and personalized medicine [74]. Wang et al*.* examined hyperbaric oxygen (HBO) therapy to enhance antitumor efficacy of Abraxane and GEM against murine pancreatic ductal carcinoma. The results revealed that HBO augmented Abraxane’s deep penetration into 3D stroma-rich spheroids composed of cancer cells and fibroblasts compared to that without HBO treatment [75]. Bhangu et al*.* investigated the use of high-frequency sound waves to transform the Dox nanodrug into a tumor selective drug molecule. In this study, breast cancer cells and fibroblast cells were used to prepare 3D spheroid model using hanging drop method and U-bottom 96 well plate. The transformed drug formed nanodrugs without requiring organic solvents which induced reactive oxygen species that selectively caused cancer cell death with only minimal cytotoxicity for fibroblasts [76].

Li et al*.* developed a combination therapy for small cell lung cancer (SCLC) using MRP1-targeted antibody-IR700 structure (Mab-IR700) for near infrared photoimmunotherapy (NIR-PIT) and liposomal Dox in a co-culture spheroid model. To simulate stromal cells and reproduce the tumor microenvironment, chemoresistant H69AR cancer cells and NIH/3T3 fibroblast cell were co-cultured to evaluate the therapeutic outcome of the combined treatment. The combined treatments showed the most efficient cytotoxic effects in vitro and greater suppression of tumor growth in vivo [77].

Darrigues et al*.* developed a 3D spheroid model to mimic heterotypic tumor-stromal composition culture of pancreatic tumor cells and hPSC to evaluate the diffusion and penetration of theranostic NPs. They observed that in monoculture models, gold NPs can flow more efficiently towards the center when compared to co-culture models, perhaps due to further corresponding necrosis and ECM. They found that cancer stroma, along with other modules such as the ECM should be considered to overcome failures in cancer therapies (Fig. 9C) [78].

Lee et al*.* investigated the potential of exosomal delivery of transforming growth factor-B receptor 1 kinase inhibitor and toll-like receptor 7/8 agonist as a combination treatment for prostate cancer. They utilized a spheroid model composed of prostate cancer cells and fibroblasts to mimic the TME for penetration study. Their results indicated that exosomes could enhance drug penetration in the 3D co-culture system [79]. Fu et al*.* explored the co-targeting of tumor stroma and c-Myc in vemurafenib-resistant melanoma cells using PEGylated liposomal formulation in a 3D co-culture spheroid model of vemurafenib-resistant cancer cells and fibroblasts. The addition of the anti-fibrotic drug Nintedanib was found to enhance the penetration of the nanocarrier into the tumor through stromal remodeling. Therefore, combined targeting of stromal components and c-Myc may be effective in treating vemurafenib-resistant melanoma cells [80].

**Fig. 10:**
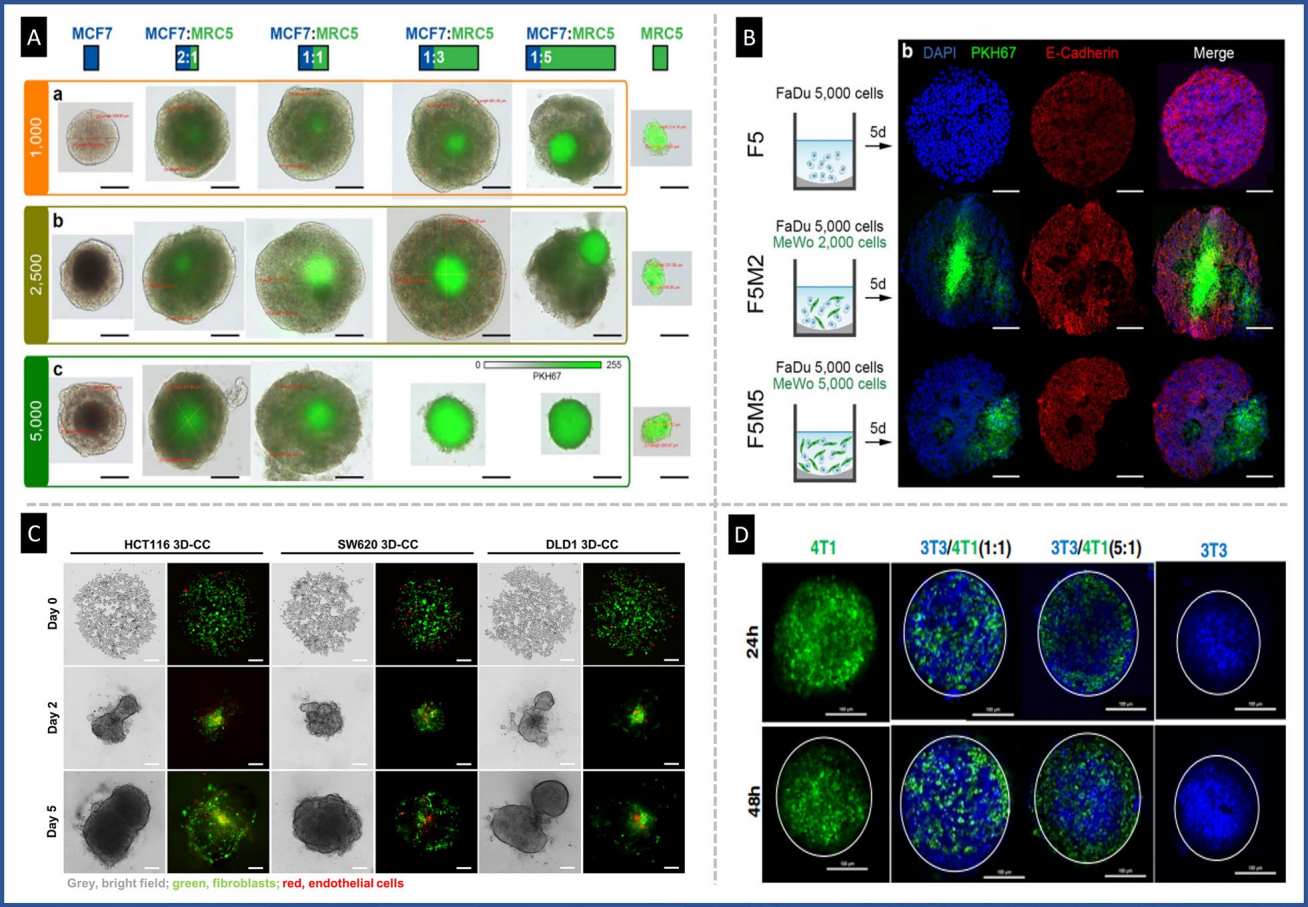
Fibroblasts distribution in heterotypic spheroid tumor models. **A** Typical optical and fluorescence images of MCF7 spheroids co-cultured with fibroblasts (MRC5) at various cell ratios. Fluorescence labeled fibroblasts were mostly condensed in the center of spheroids, reproduced from Ref. [57], under the terms of the CC BY. **B** Cryosections of monotypic (F5 tumor cells) and heterotypic spheroids at two different cell ratios in HNSCC spheroids, reproduced from Ref. [35], under the terms of the CC BY. **C** Localization of tumor cells (bright field), fibroblasts (green), and endothelial cells (red) in a tri-culture model of colorectal cancer, reproduced from Ref. [81], under the terms of the CC BY. **D** Confocal fluorescent microscopic images of monotypic and heterotypic spheroids of breast tumor cells (green) and fibroblasts (blue) representing the cellular localization in co-culture spheroids, reproduced with permission from Ref. [60], Copyright 2016 Elsevier

In another study, a heterotypic spheroid model composed of PANC-1 and CAFs was fabricated using the liquid overlay technique in 96 round-bottom well plates to evaluate bare and polyethylene glycol-modified lipid nanosystems for their ability to penetrate spheroids and transport gemcitabine as a drug model. The nanosystems were found to be more efficient than free gemcitabine in 2D culture, although this effect was lost in the 3D tumor model. These data highlight the importance of using 3D tumor models instead of 2D systems as a more realistic tool for accurate in vitro assessment of nanomedicines [76]. Figure 10 demonstrates the distribution of fibroblasts in heterotypic spheroids at different ratios of cancer cells to fibroblasts is demonstrated.

In summary, fibroblasts, particularly CAFs, are critical components of the tumor stroma due to their ability to promote tumor progression and alter the sensitivity of cancer cells to treatment. The spheroid stroma presents a significant barrier for NP penetration. Thus, tumor-stroma interactions should be considered in the modeling of tumor response to therapies. Based on the reported data, fibroblast-containing co-culture spheroids induced the production of ECM components such as fibronectin, collagen, and glycosaminoglycans, which are not easily detected in monoculture spheroids. Therefore, the inclusion of fibroblasts and CAFs in 3D spheroids may lead to more accurate evaluations of penetration, toxicity, invasion, and migration of nanomedicines into tumors. We believe that future development of this tumor model will provide a more clinically relevant tool for testing and screening novel anti-cancer formulations with improved predictive value.

#### Immune-tumor cell interaction

As previously mentioned, the interaction between tumor cells and other cell types within the TME has a significant impact on disease progression and development. In vitro models, including immune cells and other TME cells, such as macrophages, have attracted increasing attention for investigating the efficacy of new therapeutics and assessing the impact of immune cell infiltration versus tumor growth.

Macrophages have been identified as crucial players in the TME, regulating tumor initiation, progression, and metastasis [82]. TAMs derived from tissue-resident macrophages or from circulating monocytes that upon recruitment, penetrate into the cancer tissue, and consequently differentiate into M2 macrophages in response to stimuli from the TME [83, 84] and directly influence tumor invasion, angiogenesis, and ECM remodeling. Clinical studies have shown that high infiltration of TAMs in breast cancer tumor stroma is associated with poor prognosis [85].

To study immune-cancer cell interactions, Yuan et al*.* introduced a heterotypic spheroid in a microfluidic system to investigate the impact of TAMs and the stiffness of ECM on tumor cell migratory behavior [86]. The microfluidic device permitted quick establishment of breast co-culture spheroids and modulation of the TME via maintaining the spheroids in 3D collagen matrix with different stiffness, in the existence or lack of TAMs. This 3D heterotypic spheroid revealed that the complex cross-talk between TAMs and non-cellular constituents of the TME promotes cancer cell migration. LV et al*.* established a co-culture spheroid model composed of A549 cancer cells and T-lymphocyte Jurkat cells to assess anti-tumor immunity. They developed an aptamer-functionalized targeted siRNA delivery system to facilitate immune system activation and tumor progress via programmed death ligand 1 (PD-L1) inhibition, resulting in improved anti-tumor immunity and tumor growth inhibition. The constructed delivery system, NP@apt, composed of siRNA for PD-L1 and lipofectamine 2000 fused with nanovesicles derived from erythrocyte membrane modified with targeted AS1411 aptamer. The results indicated improved antitumor immunity and tumor growth inhibition [87]. Ramos et al. established a tri-culture spheroid model of colorectal cancer (CRC) composed of epithelial tumor cells, fibroblasts, and monocytes/macrophage. This model mimicked several tumor characteristics, including spatial organization, generating ECM, and central necrosis [88]. The model was investigated for combined chemoimmunotherapy impacts of spermine-modified acetalated dextran NPs loaded with the chemotherapeutic Nutlin-3a (Nut3a) and granulocyte–macrophage colony-stimulating factor (GM-CSF) as an immunomodulator. While NPs were effectively taken up in the 2D monoculture model, a substantial reduction was observed in the spheroid model, although NPs induced an anti-proliferative influence in both 2D and 3D spheroid models. Furthermore, Nut3a was shown to moderately alter the polarization of macrophages in the heterotypic 3D model towards an anti-tumor M1-like phenotype. Almeida et al*.* evaluated the anticancer efficacy of camptothecin-loaded micelle in a tri-culture of monocytes, cancer cells, and human fibroblast, with significant effects observed on metabolic activity and spheroid size reduction [89].

In addition to heterotypic spheroid models of cancer cells with fibroblasts and immune cells, other co-culture models have also been investigated. Sokolova et al*.* proposed a heterotypic 3D culture of spheroids composed of human primary astrocytes, pericytes, and brain endothelial cells that demonstrate reproducible neurovascular unit features and functions [90]. They synthesized fluorescent ultrasmall surface-functionalized gold NPs as carriers for imaging/drug delivery into brain cells and investigated their function on 3D co-culture spheroids.

#### Endothelial-cancer cell interaction

**Fig. 11:**
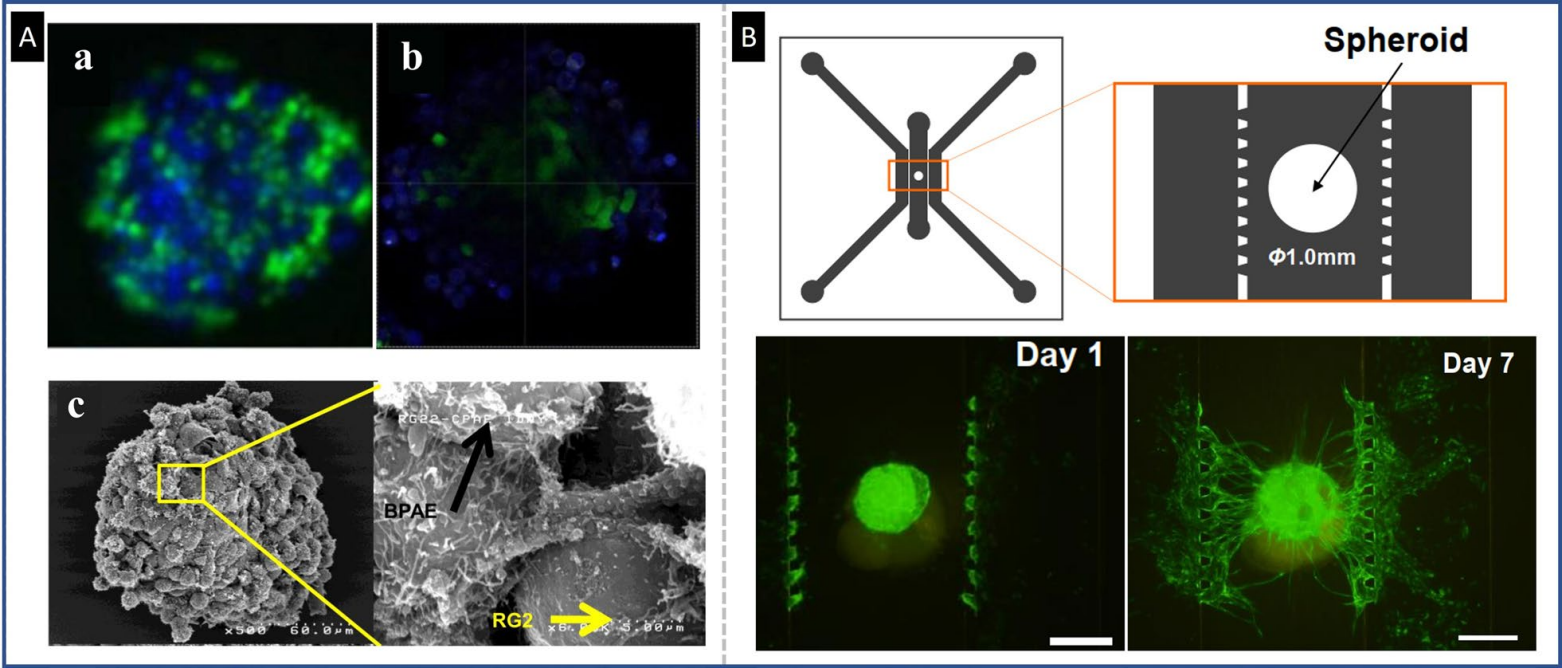
Application of the heterotypic spheroid with endothelial-cancer cell interaction: **A** Epifluorescence images of BPAE-RG2 co-cultured spheroid during initial assembly after 24 h of spheroid formation, fluorescent confocal cross-section image reveals that BPAE cells (blue) have completely covered the RG2 core (green) (a and b). Micrographs taken using the SEM show a BPAE-RG2 spheroid with BPAE cells bonded onto RG2 cells (c), reproduced from Ref. [34], under the terms of the CC BY. **B** The layout of a microfluidic device for modelling the vascular network, as well as fluorescence microscopy images of the stages of vascular network formation originating from tri-cultured spheroids in the chip. The scale bar represents 500µm. On first day, a tri-culture spheroid that was comprised of GFP-HUVECs was put in the center chamber of the microfluidic platform. Moreover, at that time, the GFP-HUVECs were cultured into the microslit channels. Tiny angiogenic sprouts grew in the tumor spheroids and microchannels within the first days. Over time, the angiogenic sprouts branched out and elongated as though they were attracted to one other. Finally, as can be seen, a vascular network has been formed on the seventh day, Reproduced with permission from Ref. [93], Copyright 2022 Elsevier

Endothelial cells (ECs) play a crucial role in tumor angiogenesis and invasiveness, making them a prime target for antitumor therapies. Recent advances in heterotypic spheroid culture methods have enabled EC-targeting approaches. For instance, a 3D co-culture spheroid model composed of tumor, endothelial, and fibroblast cells has been developed, and the anticancer impact of NPs conjugated to Anginex for targeting ECs in the model has been studied (96). Ho et al*.* investigated the use of iron oxide NPs conjugated with tumstatin peptide, which is highly expressed on the cell surface of neovascular ECs, to target tumor vasculature in a novel endothelial-coated spheroid model. The co-culture spheroids successfully mimicked the physiological circumstances with a leaky endothelium layer around a core of glioma tumor cells (Fig. 11A). The tumstatin-iron oxide NPs demonstrated penetration and specific targeting to the ECs coating the tumor in the 3D model and resulted in approximately twofold larger uptake in vitro and 2.7-fold greater tumor neo-vascularization suppression [34]. Widjaya et al*.* established a 3D spheroid model composed of tumor cells, RAW264.7, and human umbilical vein endothelial cells (HUVECs) to evaluate the uptake, cytotoxicity, and anticancer effects of PTX-loaded corosolic acid liposomes (PTX/CALP). The smart PTX-loaded liposomal formulations based on CALP were able to overcome the harsh tumor biological barriers, increase the induction of immunogenic cell death, and achieved overall acceptable impacts [91]. To better replicate the TME aspects of breast cancer in vitro, a 3D co-culture spheroid was developed using the hanging drop technique. The 3D model was obtained by integrating tumor cells, ECs, and fibroblast cells for optimization and internalization of NPs for drug delivery and to monitor biological response to radiation. The group demonstrated progress in preclinical evaluation of existing and innovative cancer nanotherapies [92]. In another study, Chantarasrivong et al*.* evaluated the disposition of liposomal formulations targeting E-selectin in tumor spheroids for assessing tumor vascular-targeting drug delivery systems. These spheroids with perfusable vascular networks have been established in a microfluidic system to resemble in vivo situations (Fig. 11B). The heterotypic HUVECs/lung fibroblast spheroids can develop angiogenic sprouts and produce a continues vascular network of HUVECs, which are seeded in microfluidic channels of the device [93].

### Screening photothermal/photodynamic therapies

Co-couture spheroid models have been employed in various cancer therapies, including candidate cancer therapies, screening of chemotherapeutics, chemotherapeutics combinations, and immunotherapies. Heterotypic platforms are also used in photothermal/photodynamic therapies as they offer more accurate microenvironments, allowing scientists to consider spatiotemporal oxygen gradients and cancer cell adaptations [94].

Photothermal therapy (PTT) uses a photothermal (PT) agent to convert photoenergy into heat for the thermal ablation of cells. It is minimally aggressive yet efficient approach to cancer therapy. In photodynamic therapies (PDT), light-sensitive drugs called photosensitizers produce reactive oxygen species through a series of photochemical reactions [95]. Consequently, the generated oxidative stress can induce intracellular lipid peroxidation, DNA injury, and protein damage, eventually leading to cell death. In recent years, nano-based encapsulation has significantly improved the photodynamic features of photosensitizers. The encapsulation procedure can improve the solubility of drugs with poor water solubility, enhance the specificity of photosensitizers delivery to target cells, and reduce issues concerning photodegradation of photosensitizers. Nanocarriers can also provide controlled release of drug. A growing number of new-generation photosensitizers, primarily based on NP-based PTT/PDT have shown great potential as cancer therapy [96]. To investigate the cross-talk between developing tumors and the neighboring stroma, Emami et al*.* prepared 3D breast cancer spheroids combined with M2-like macrophages (M2-M) to simulate in vivo environments [97]. Since TAM with the M2 phenotype are fundamental suppressors of anticancer treatments and play an important role in chemoresistance in most solid tumors. The group also assessed the efficiency of cetuximab (Cmab)-conjugated GNR upon photothermal therapy with NIR laser to demonstrate the effectiveness of their nanosystem in an aggressive breast cancer model with M2-M. The effectiveness of Cmab-GNR plus NIR in overcoming chemoresistance in high TAM-infiltrated 3D spheroid environments highlights the potential applications of photothermal-based nanotherapeutics to address chemoresistance in solid tumors. Park et al*.* developed a dextran sulfate-based nano-photosensitizer to target M2-like TAMs for enhanced PDT. To evaluate the efficacy of the system, they developed a heterotypic spheroid of 4T1 tumor cells/macrophages [94]. The nano-based photosensitizer successfully induced apoptosis in tumor cells in co-culture spheroids under laser irradiation. Piehler et al*.* generated heterotypic spheroids of pancreatic tumor cells and fibroblast [98]. The results showed that identical seeding quantities of cancer cells and fibroblast could form compact spheroids containing collagen fibers after 7 days in culture in the absence of exogenously added ECM elements. It was also investigated whether mild hyperthermia using iron oxide NPs could alter the collagen fiber architecture in the generated 3D co-culture pancreatic tumor model. Yu et al*.* prepared a heterotypic tumor spheroid of pancreatic cancer cells and fibroblasts (Pan02/NIH 3T3) [99]. Due the significant role of CAFs in the pancreatic tumor stroma, new thermosensitive liposomal formulations were developed that are responsive to CAFs. These liposomes were encapsulated with albumin NPs of PAX (HAS-PTX) and integrated with IR-780, a photothermal agent, into the shell of the liposomal formulation for PTT. The CAF-responsive liposomes increased the drug retention of HAS-PTX in TME, leading to disruption of the stromal barrier and greater local drug accumulation at the tumor region. Consequently, under stimulation of NIR laser irradiation, IR-780 generated hyperthermia that destroyed cancer cells and promoted the release of tiny HAS-PTX in the deep tumor sections.

**Fig. 12:**
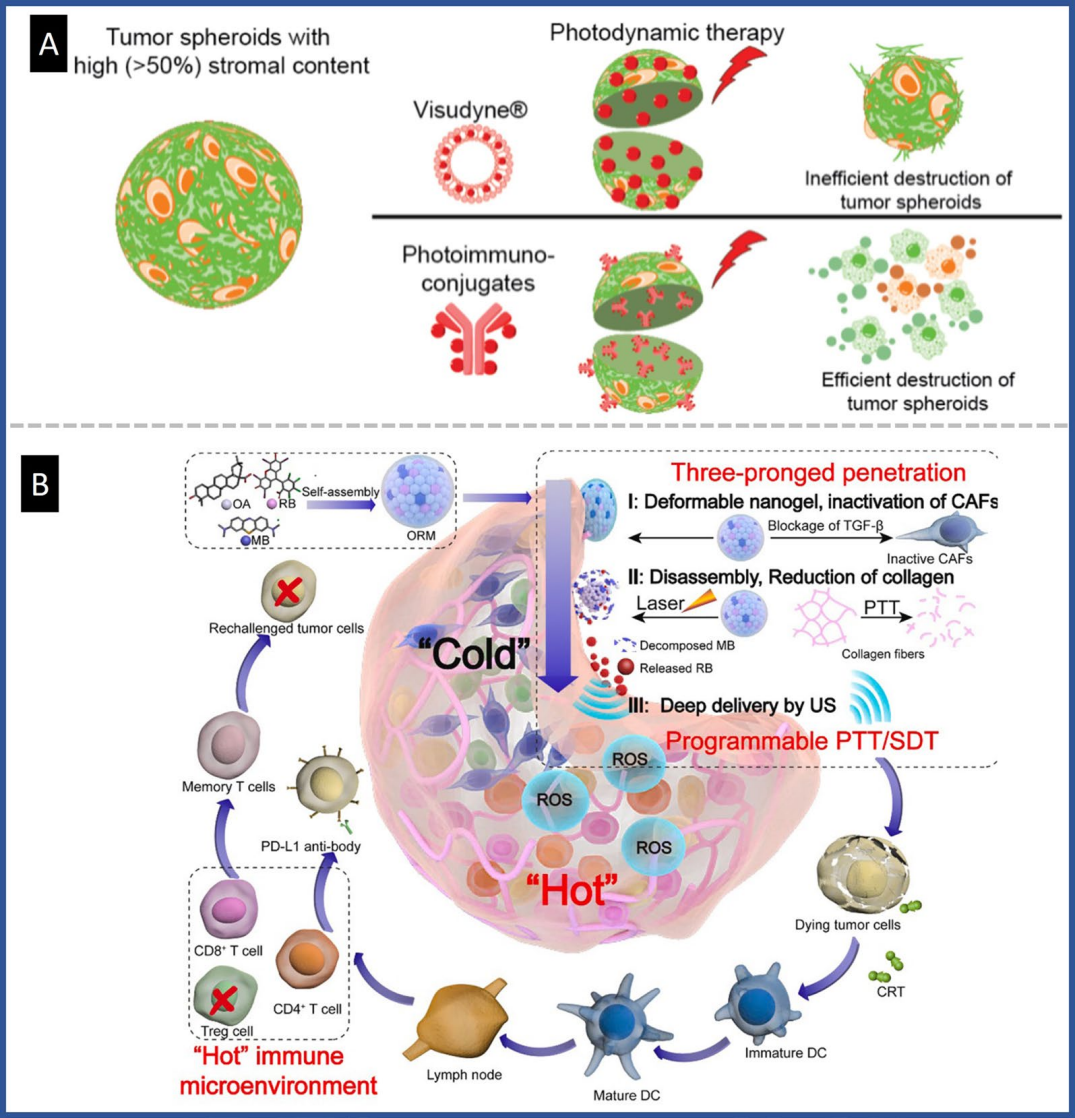
Application of co-culture spheroid model to evaluate drugs with PDT property **A** Schematic figure of the results of Visudyne and PIC performance on co-culture spheroids with stromal cell content above 50% Reproduced with permission from Ref. [101], Copyright 2022 American Chemical Society. **B** Diagram of the three-branched penetration of ORM into tumors for switchable PTT/SDT-assisted anti-PD-L1 immunotherapy, Reproduced with permission from Ref. [102], Copyright 2022 Elsevier

Mesenchymal stem cells (MSCs) have been suggested as a promising carrier for nanomedicine due to their natural affinity for the TME. However, the preclinical evaluation of MSCs is still in its early stages, largely due to the lack of available platforms for testing these therapeutics. To address this issue, Ferreira et al*.* established a 3D breast tumor spheroid model composed of breast tumor cells and CAFs to mimic the dense structure of breast tumors with central necrosis. This model was used to evaluate the ability of MSCs to deliver multi-modal theranostic NPs to breast tumor cells [100]. The chosen therapy was a chemo-photothermal combination, using melanin-biomimetic NIR-light responsive polydopamine (PDA) NPs loaded with Dox and indocyanine green (ICG), a NIR fluorescence imaging probe that could also be used as a photothermal agent. The study found that MSCs possess tumor-homing properties and can be used as Trojan-horse structures to deliver multi-functional NPs to physiomimetic breast co-culture spheroids. To investigate the direct impact of stromal content on the responsiveness of PDAC spheroids to PDT and antibody-targeted PDT (PIT), Saad et al*.* developed PDAC co-culture model with varying proportions of patient-derived CAFs [101]. The efficacy of PIT using cetuximab photoimmunoconjugates (PICs) of benzoporphyrin derivative (BPD) was compared with the clinically approved liposomes of BPD (Visudyne^®^). The results showed that while Visudyne^®^-PDT and PIT were efficient in a co-culture model with low stromal content, as the stromal content increased, the effectiveness of Visudyne^®^-PDT was reduced by up to tenfold although no alternation in PIT efficacy was observed. This was attributed to the ability of PICs to accumulate inside spheroids with higher stromal content, while Visudyne^®^ was restricted to the outer shell of spheroids. These findings are particularly important for determining the optimal therapy for tumors with high stroma content (Fig. 12A).

The study by Yakavets et al*.* aimed to recreate the TME of head and neck squamous cell carcinoma by co-culturing patient-derived human granular fibroblasts (as CAFs) with pharynx squamous cell carcinoma in a spheroid model [35]. They investigated whether a stroma-rich TME affected the accumulation and efficacy of PDT in nano-based lipid formulations. According to their findings, the stromal microenvironment significantly altered the uptake of NPs, but the efficacy of PDT was not affected by the presence of stromal constituents. Lee et al*.* developed two co-culture systems, including HeLa/HUVEC spheroids to model tumor/endothelium and HeLa/Ovarian heterotypic culture to mimic a metastatic-based secondary tumor [35]. They evaluated the anticancer effects of a hybrid gold NP-functionalized graphene oxide (Au@GO) for PTT in spheroids for tumor screening applications. Li et al*.* developed a heterotypic spheroid model of triple negative breast cancer cells and fibroblasts to evaluate self-assembled nanoregulator ORM based on oleanolic acid (OA) for simulating the TME and investigated the efficiency of drug delivery into central part of tumor for combined PTT and immunotherapy. They used methylene blue as photothermal agent and rose bengal (RB) as a sonosensitizer, which combined with OA via electrostatic interactions to achieve ORM nanogel. While OA caused fibroblast inactivation by blocking the TGF-B receptor, physical tumor therapy could remodel ECM, allowing additional penetration of RB. Therefore, ultrasound can promote the deep penetration of RB for efficient sonodynamic therapy (SDT) [102] (Fig. 12B).

Flont et al*.* aimed to develop a novel perfusion Lab-on-a-chip platform for co-culturing non-tumor and ovarian tumor cells [103]. They offered an innovative 3D tumor model that simulates the organization of a heterogeneous tumor tissue. According to the results, the use of free meso-tetrafenylporphyrin and nanoencapsulated meso-tetrafenylporphyrin could considerably increase the effectiveness of PDT in managing ovarian cancer. Additionally, the newly developed 3D cellular model was shown to be suitable for rapid screening of anticancer agents and has potential for use in personalized medicine. In a similar study, Zuchowska et al*.* demonstrated a 3D co-culture tumor model under microfluidic conditions that effectively mimicked the TME of tumors [104]. They utilized the co-culture system to assess the efficiency of PDT with a nanoencapsulated photosensitizer (meso-tetraphenylporphyrin, nano-TPP). Based on the results, the heterotypic spheroid displayed higher resistance to PDT procedures compared to the spheroid monocultures.

However, planning studies for the application of heterotypic spheroids for nanoparticles/drugs evaluation poses various challenges, such as the selection of appropriate cell types, determination of suitable cell ratios, and choice of a proper method for spheroid production. Table 1 summarizes important information from studies that have utilized the heterotypic spheroid model to investigate the safety or efficacy of nanoparticles/drugs.

**Table 1:** The co-culture spheroid production methods along with the utilized nanoparticles/drugs and the obtained results were demonstrated.

Types	Co-culture approach	Spheroid formation method	Tumor/somatic cells Ratio	Scaffold	Drug/nanoparticle	Nanoparticle type	Main finding	Refs
Fibroblast	Direct-Mixing	Liquid overlay (ULA)	NCIH460/WI-38 (1:1) A549/WI-38(1:1)	Gelatin	Paclitaxel/ Eudragit®RL 100	Polymeric nanoparticle	Paclitaxel -ENP reduced spheroids' invasiveness. WI38 cells increased spheroids' Paclitaxel resistance	[69]
	Direct-Mixing	Liquid overlay (Microstamped petri dishes coated with Pluronic F-127)	4T1/ NIH3T3 (1:1 &5) BJ-hTERT/MDA-MB-231 (1:5) hPSC/Panc-1 (1:5)	NO	Cy5- PLGA NPs PLGA NPs silica NPs	Silica nanoparticle	NP penetration decreased in co-culture spheroid, whereas α-SMA and e-cadherin expression increased and decreased, respectively	[60]
	Core shell	Liquid overlay (Insphero plate) and hanging drop system	BeWo/HVMF (core)	NO	CdTe-COOH NPs CuO NPs	Copper oxide nanoparticle	HVMFs formed a stable core of the co-cultured spheroid surrounded by BeWo cell layers	[105]
	Direct-Mixing	Liquid overlay (agarose coated plate)	panc-1/WI-38 (1:1)	NO	Iron oxide NPs	Iron oxide nanoparticles	Nanotoxicity of NPs in co-cultured spheroids increased with temperature. 7-day co-culture spheroids have collagen fibers	[98]
	Direct Mixing	Liquid overlay, coted plate	FaDu cells/MeWo cells (1 & 5:1)	No	3 liposomes based on temoporfin for PDT(foslip, cyclodextrin liposome and extra vesicle)	Liposome	Temoporfin-EV had remarkable overall cellular absorption as well as PDT efficiency that was superior to other NPs	[35]
	Direct Mixing	Microwell-based microfluidic	MCF-7/HMF (1:1)	No	Nano-TPP for PDT	micelle	Co-culture spheroids are more resistant to PDT than monocultures	[104]
	Core-sheell	Liquid overlay (agarose coated plate)	BxPC-3(core)/NIH-3T3	No	Acid-sensitive, membrane-disruptive micelle (M-14K)	micelle	Treatment with NPs caused stroma remodeling and cancerous cell elimination simultaneously	[62]
	Layer by layer	Not spheroid Transwell culture	A2780/HOF (1:3)	NO	nano-TPP for PDT	micelle	HOF cells stimulate the proliferation of A2780. The nano-TPP is more selective than free TPP	[103]
	Direct mixing	Modified liquid overlay in ULA	MDA-MB-231/BCAFs (1:2)	NO	PDA-ICG-DOX administrated as nano-in-cell (hBM-MSCs) antitumor therapy for PTT	polymeric nanoparticles	hBM-MSCs loaded PDA-ICG-DOX adhere to spheroids, and showed higher antitumor potential vs. PDA-ICG-DOX	[100]
	Direct mixing	Liquid overlay (Agarose micromold)	MCF-7/NHDF (1:1)	NO	SLN-Mito	Lipid based nanoparticle	SLN-Mito could penetrate to spheroids and showed higher anti-cancer efficacy with compare to the Mito alone	[65]
	Direct mixing	Liquid overlay (agarose coated plate)	LS/hSkMC (1:1) LS/hDFa (1:1)	NO	Cu(DDC)2 liposomes	Liposome	The administration of Cu(DDC)2 liposomes significantly decreased the vitality of co-cultured spheroids	[72]
	Direct mixing	Liquid overlay (matrigel coated plate)	MDA-MB-468/NIH-3T3 (2:1)	Matrigel	Oligosaccharide library of heparin sulfate	Other	The potential of HS-based nanovehicle for EGFR targeted cancer therapy even in the presence of fibroblast layer was exhibited	[63]
	Direct mixing	Not mentioned	4T1/NIH-3T3 (4:1)	Matrigel	NE-LMP	Liposome	NE-LMP may efficiently target and breakdown ECM in co-cultured spheroids, Increased PTX and NPs penetration into the spheroid was observed	[68]
	Direct mixing and core–shell preparation	Liquid overlay (ULA)	Direct: UMUC3/NIH-3T3 (2:1) Core–shell: UMUC3(core)/NIH-3T3	NO	LCP-NPs	Other	The layer of fibroblasts acted as the principal barrier to the penetration of LCP-NPs, and NPs were shown to accumulate in the shell of the co-cultured spheroid	[66]
	Direct mixing	Liquid overlay (poly-hema-coated	A549/MRC5	No	Dox-loaded nanoMOFs	Metal organic framework	DOX was successfully encapsulated into nanoMOFs, distributed throughout the spheroids and uptake across the cells, but was prevented from entering the nucleus by being trapped in the lysosome	[67]
	Direct mixing	Liquid overlay (ULA)	MIA PaCa-2 + PCAFs (5000 cell) (0 to 90% of PCAFs)	No	Visudyne-PDT	Liposome	Visudyne-PDT was efficient in co-culture spheroids with low stromal content but less effective in high-stromal ones	[101]
	Direct mixing	Liquid overlay (ULA)	Pan02/TGF-β activated NIH 3T3 (2:1)	No	HSA-PTX@CAP-ITSL therapy for PTT	Polymeric nanoparticle	Nanodrug well designed for combinatorial therapy, chemotherapy with PTT, for PDAC treatment	[32]
	Direct Mixing	Hanging drop	SKOV-3/MRC-5 (1:1) SKOV-3/activated MRC-5 (1:1)	No	Coumarin-6 encapsulated by PLGA NPs	PLGA nanoparticle	Introducing the new matrix-embedded co-culture spheroid for modeling the ovarian ascites and metastatic environment	[64]
	Direct Mixing	Liquid overlay (ULA)	UM-SCC-1/NHLF (1:1)	No	miR-34a-HDL NPs for Radiotherapy	Polymeric nanoparticle	In 3D spheroids, but not 2D culture, miR-34a interferes with the G2/M cell cycle arrest of UM-SCC-1 cells that was induced by radiation	[73]
	Direct Mixing	Microencapsulation	AsPC-1/PSCs (1:3) PANC-1/PSCs (1:3)	Alginate	SiO2-based microparticle pH sensor	Silica based nanoparticle	The pH of the extracellular environment was different in 3D co-cultures of AsPC-1 and PSCs than in mono-cultures of either cell type	[74]
	Direct Mixing	Hanging drop	Panc02/NIH-3T3 (2:1)	methylcellulose	Abraxane	Polymeric nanoparticle	Hyperbaric oxygen condition enhanced the deep penetration of Abraxane in a 3D stroma-rich spheroid	[75]
	Direct Mixing	Liquid overlay (poly-hema-coated)	PANC-1/CAF08 (1:4)	No	Polyethylene glycol-modified lipid nanosystems for gemcitabine	Lipid based nanoparticle	Nanodrugs were more effective than free drugs in a 2D tumor model, but their effectiveness was lost in a 3D tumor model	[106]
	Direct Mixing	Liquid overlay (ULA)	A375R/dermal fibroblast (2:1)	No	ARNIPL targeting the	PEGylated liposome	ARV + Ni inhibited tumor growth better than free drugs. Greater liposome penetration may clarify why ARNIPL inhibits tumors more than free ARV + Ni	[80]
	Direct Mixing	Liquid overlay (ULA)	Panc-1/PSCs (1:1, 1:2, & 5:1)	No	Functionalized AuNRs for Multimodal imaging	Gold nanoparticle	Analyses of PTM and PA showed that the NPs did not penetrate far into the stroma of the large (> 500 um) spheroid	[78]
	Direct Mixing	Liquid overlay (ULA)	CCD-1059-SK)/PC-3 (1:1)	No	Drug-loaded EXOs with targeting	Liposome	EXOs showed excellent co-cultured spheroid penetration	[79]
	Direct Mixing	Hanging drop	BT474/ 3T3 (1:1)	No	Doxorubicin nanodrug	Other	Penetration, cytotoxicity, and selectivity of the nanodrugs validated in co-cultured spheroid	[76]
	Direct Mixing	Not mentioned	4T1/NIH3T3 (1:5)	matrigel	Nanoregulator ORM for PTT and SDT	Other	The EPR effect and flexibility of ORM nanogel allowed it to easily penetrate into co-culture spheroids	[102]
	Direct mixing	Liquid overlay (ULA)	H69AR/NIH-3T3 (1:1)	No	Mab-IR700 and Doxil for NIR-PIT and targeting	Liposome	In co-culture spheroids, the highly increased permeability and retention effect caused by the combination therapy was found	[77]
Endothelial	Direct mixing	Liquid overlay (Agarose micromold)	HUVEC/hDPSC (1:1)	No	O2-sensitive nanoparticles	Polymeric nanoparticle	Co-cultured spheroids had lower respiration activity due to strong glycolysis and pentose-phosphate pathways. Therefore, they had higher peripheral and core oxygenation than monoculture spheroids	[107]
	Direct mixing	Liquid overlay (Agarose micromold)	HeLa/Ovarian HeLa/HUVEC	No	Doxorubicin-loaded Au@GO for PTT	Graphene and Gold based nanoparticle	HeLa/HUVEC spheroid serves as a tumor/endothelial model, and HeLa/Ovarian spheroid can be utilized to imitate a secondary tumor produced by metastasis	[108]
	Direct Mixing	Liquid overlay (Agarose micromold)	RG2/BPAE (1:1)	No	Tumstatin-Fe_3_O_4_ NPs	Iron oxide nanoparticle	RG2 produced a spheroid core and BPAE cells were grown on it. Tumstatin-Fe3O4 NPs penetrated endothelial cell-coated spheroid	[34]
Immune cell	Direct mixing	Liquid overlay, AggreWell	MDA-MB-231/M2-M (10:1)	Matrigel	Cmab-GNR for PTT	Gold nanoparticle	Cmab-GNR with NIR were successful to overcome the high-EGFR overexpressing chemoresistant triple-negative breast cancer model	[97]
	Direct Mixing	Liquid overlay (poly-hema-coated)	A549/T-lymphocyte Jurkat cells	No	NP@apt with targeting	Lipid based nanoparticle	In treated co-cultured spheroid, NPs@apt may limit A549 cell PD-L1 protein expression, block PD-1/PD-L1 immune escape and activate T cells to slow tumor growth	[87]
	Direct mixing	Liquid overlay (ULA with methylcellulose)	B16F1 and B16F10/ THP-1 (1:1)	No	SNA/hSPION-Au for targeting and PTT	Gold based nanoparticle	SNA/hSPION-Au increased macrophage infiltration in co-culture spheroids	[109]
	Core–shell	Liquid overlay (agarose-coated)	4T1 (core)/ Macrophage	No	DS-Ce6 for PDT and targeting	Other	In co-cultured spheroids, under laser irradiation, DS-Ce6 was able to trigger apoptotic cell death, and it targeted M2-like TAMs inside the tumor tissue	[110]
Triple	Direct mixing	Liquid overlay (agarose micromold)	Monocytes/HCT116/HIF (4:1:4)	No	Polymeric NPs of Sp-AcDEX	Polymeric nanoparticle	Epithelial cells took up nanoparticles in 2D models but rarely in 3D ones	[88]
	Direct-Mixing	Liquid overlay (ULA)	hpBECs/hpPs/hpAs (1:1:1)	NO	Au-Click-FAM, Au-Click-Cy3 and Au-CGGpTPAAK-FAM	Gold nanoparticle	Used the co-culture model as BBB model: core is mostly comprised hpAs cells that covered with hpPs, and hpBECs form the outermost layer. Within this model, GNP have the potential to act as a carrier system for both biomolecule[90]s and dyes	[90]
	Layer by layer	Not spheroid Transwell culture	NOF/HPMC/CaOV3 or CaOV3CisR	No	PDMAEMA–BSA conjugate for carrying siRNA	Polymeric nanoparticle	An organotypic model of high-grade serous ovarian cancer to assess the anti-metastatic potential of Wnt receptor ROR2 targeting Polyion complex NPs was created	[111]
	Direct mixing	Liquid overlay (poly-Hema-coated	PANC-1/coated MRC-5/HUVEC (1:9:4)	Fibronectin or gelatin	Dox-loaded polymer NPs	Polymeric nanoparticle	Using the LSFM based 3D tomography, reaching the DOX to the core of co-cultured spheroid was confirmed, but it was not confirmed for Dox-loaded polymer NPs	[70]
	Direct mixing	Hanging drop	4 T1/RAW264.7 /HUVEC (4:1:1)	No	PTX/CALP and PTX/LP	Liposome	The efficacy of PTX/CALP NPs was comparable to free drug and significantly higher than PTX/LP duo to CALP's high uptake and the synergy of CA and PTX	[91]
	Direct Mixing	Liquid overlay (PDMS micromold)	Monocytes/HCT116/HIF (4:1:4)	No	CPT-loaded micelles	Micelle	NPs had a substantial influence on the biochemical activity and size reduction of the spheroid	[89]
	Direct Mixing	Liquid overlay (ULA)	MCF-7/nhLF/HUVECs (1:3:1)	No	3′-CE SELEX mimic	Liposome	Triculture spheroid was used in a microfluidic device to generate a perfusable vascular network for evaluating tumor-targeted nanodrug delivery systems	[93]

At the end of the table, there is a guide to abbreviations, which are arranged in alphabetical order within each category

Tumor cells: Mouse breast cancer (4T1), human ovarian cancer cells (A2780), human lung cancer cells (A549), Human pancreatic cancer cell line (AsPC-1), Murine melanoma cell lines (B16F1 and B16F10), human placental choriocarcinoma (BeWo), human ovarian cancer cell line (CAOV3), cisplatin-resistant CaOV3 cell line (CaOV3CisR), human pharynx squamous cell carcinoma (FaDu), human small cell lung cancer cell line (H69AR), human colorectal carcinoma (HCT116), head and neck squamous cell carcinoma (HNSCC), neuroblastoma cell line (LS), human breast adenocarcinoma cells (MCF-7), Triple negative breast cancer derived cell line (MDA-MB-231), The human pancreatic ductal adenocarcinoma cell line (MIA PaCa-2), murine model of pancreatic cancer (Pan02), pancreatic carcinoma of ductal cell (Panc-1), human prostate cancer cell line (PC-3), Pancreatic ductal adenocarcinoma (PDAC), Human immortalized pancreatic stellate cells (PSCs), Rat glioblastoma cell line (RG2), human ovarian cancer cell line with epithelial-like morphology (SKOV-3), head and neck cancer cell line (UM-SCC-1), human bladder cancer (UMUC3).

Fibroblast cells: human breast cancer-associated fibroblasts (BCAFs), cancer associated-fibroblasts (CAF08), human fibroblast cell line (CCD-1059-SK), primary human dermal fibroblasts, and adult (hDFa), Human intestinal fibroblast (HIF), human mammary fibroblasts (HMF), human ovarian fibroblasts (HOF), Primary human villous mesenchymal fibroblasts (HVMF), human primary astrocytes (hpAs), human primary mesothelial cells (HPMC), human primary pericytes (hpPs), primary human skeletal muscle cells (hSkMC), human fetal lung fibroblasts (MRC-5), granular fibroblasts, derived from human melanoma (MeWo), Human Lung Fibroblasts (NHLF), Mouse embryonic fibroblast (NIH-3T3), human primary fibroblast cells (NOF), primary pancreatic cancer-associated fibroblasts (PCAFs), human embryonal lung fibroblast cell line (WI-38).

Endothelial cells: bovine-pulmonary arterial endothelial (BPAE), human primary brain endothelial cells (hpBECs), human umbilical vein endothelial cells (HUVEC)

Immune cells: primary human monocyte (monocyte), mouse monocyte macrophages (RAW264.7), immortalized human monocytes (THP-1)

Stem cells: human bone-marrow derived MSCs (hBM-MSCs), Human Dental Pulp Stem Cells (hDPSC), mesenchymal stem cells (MSCs),

Nanoparticles and chemicals: Nintedanib co-loaded PEGylated nanoliposomes (ARNIPL), Gold nanorods (AuNR), 3’-(1-carboxy)ethyl sialyl Lewis X mimic (3′-CE SLEX mimic), Corosolic acid liposome (CALP), CdTe surface-functionalized with carboxylate moieties (CdTe-COOH), Cetuximab-anchored gold nanorod (Cmab-GNR), Camptothecin -loaded micelles (CPT-loded micelles), Cy-5-conjugated PLGA nanoparticles (Cy5-PNPs), Diethyldithiocarbamate (DDC), doxorubicin (dox), Liposomal doxorubicin (Doxil), N,N-dimethyl amino ethyl methacrylate (DMAEMA), Dextran sulfate-conjugated chlorin e6 (DS-Ce6), Eudragit® RL 100 nanoparticles (ENP), Exosomes (EXOs), Fibroblast Activation Protein (FAP), gold nanoparticle (GNP), high-density lipoprotein NPs (HDL NPs) lipid-coated calcium phosphate nanoparticles (LCP-NPs), MRP1-targeted antibody-IR700 system (Mab-IR700), Acid-sensitive, membrane-disruptive micelle (M-14K), Doxorubicin nanodrug (NDDOX),Chimeric cell membrane proteins and membrane-bound elastase (NE-LMP), gelatin methacryloyl (matrigel), nanoscale iron carboxylate metal–organic frameworks (nanoMOFs), Nanoencapsulated meso-tetraphenyl porphyrin (nano-TPP), polydopamine nanoparticles loaded with indocyanine green-doxorubicin combinations (PDA-ICG-DOX), Programmed death ligand 1 (PD-L1), Poly(D,L-lactic-coglycolic acid) (PLGA), Paclitaxel (PTX), mitoxantrone-loaded solid tumor nanoparticles (SLN-Mito),* Sambucus Nigra* agglutinin (SNA)-conjugated hollow goldiron oxide nanoparticles (SNA/hSPION-Au), Polyethylene glycol-modified lipid nanosystems for gemcitabine (SNs_PEG), Spermine-modified acetalated dextran (Sp-AcDEX), Tumstatin-iron oxide nanoparticles (tumstatin-Fe3O4 NPs), clinically approved liposomal formulation of benzoporphyrin derivative (Visudyne),

Other: Blood Brain Barrier (BBB), extra vesicle (EV), Light Sheet Fluorescence Microscopy (LSFM), near infrared photoimmunotherapy (NIR-PIT), photoacoustic (PA), photothermal microscopy (PTM), sonodynamic therapy (SDT)

## Challenges, guidelines, and future directions

Here, we initially focus on the challenges concerning 3D spheroid culture establishment and application. With regard to preclinical studies for developing and screening the innovative anticancer therapeutics, it is critical to have access to spheroids with uniform size and reproducible shape [112]. In this regard, standardized procedures for tumor formation and analysis are critical. Among the various factors that influence the morphology of spheroids, the role of fabrication method is critical. Hanging drop technique is simple and the size and morphology of spheroids are reproducible, however, the whole procedure is labor intensive and time consuming. Tung et al*.* established a 384-format hanging drop array plate for spheroid generation and maintenance in culture [113]. This system is a simplified conventional hanging drop approach with tedious liquid handling procedures along with increased strength in droplet stability for long-term in vitro maintenance. Concave microwells, for example, made it easy for certain cancer cells to form uniform-sized and reproducible spheroids [114].

Microwell-based microfluidic devices also overcame certain challenges. In microfluidic devices, the size and shape of spheroids are uniform, however, the fabrication process and optimization are intricate and require customized instruments as well as trained users [115]. Mass production of size-controlled spheroids can also be achieved using microfluidic devices [116, 117].

The device is specified with several microwells connected to a loading chamber through a microchannel. Each microwell is evenly filled with a cell suspension to achieve spheroids of uniform size. Trapping cells in U-shaped microstructures likewise offers a massive and high-throughput platform for spheroid formation. Cells can be trapped by either active (using external power such as magnetical or optical sources to capture the cells) and/or passive methods [118, 119].

Bioreactors/spinner flasks are another approach for spheroid mass production, however, the non-uniformity in spheroid shape is very common in this method [120]. Thus, selecting an appropriate method for spheroid formation is critical, as fabrication approaches does not perform equivalently to generate spheroids. Besides from method of fabrication, other elements including the cell type, the medium, and the cell density can affect the spheroid size and structure [112].

Culturing tumor spheroid have several distinctive properties; they own chemical gradients of oxygen and nutrients at diameters usually from 200 μm. Once the spheroids raise to over 200 μm in size, a necrotic core may be observed since the outer layer of cells obstructs nutrient and oxygen penetration into their core [121]. Based on the purpose of the study and on the applied technique, spheroids of any dimension can be obtained. In large spheroids (starting from about 500 μm in diameter) a peripheral growing zone, an internal quiescent zone (caused by restricted availability of oxygen, nutrients, and metabolites), and a necrotic core can be observed which mimicking the cellular heterogeneity of solid tumors [10]. Standardized procedures for spheroids formation and characterization are thus required; particularly to avoid shape- and size-related heterogeneity for treatment efficiency assessment. Variable hypoxic core was also observed in spheroids based on their size [122]. This feature is important especially for examinations on hypoxia-selective cytotoxic compounds such as tirapazamine derivatives [123]. Furthermore, the spheroid size might affect treatment assessment.

Several other challenges remain to be addressed for spheroid models. The lack of simple organized method for rapid homogenous evaluation of cellular responses is one of key reasons that 3D spheroids have not adapted for drug screenings. Molecular tests such as western blotting and RT-PCR are tough to accomplish on spheroids due to low number of cells available within spheroids. Nonetheless, microscale versions of western blot have currently been established that can be performed with limited number of cells [124–126]. Quantitative RT-PCR (qRT-PCR) evaluation is however, a challenge due to spheroids RNA extraction inefficiency. The cell lysates obtained from at least 2000 cells per sample can be purified using specifically designed RNA extraction kits, which can be subsequently used for gene expression analysis [120]. What's more, histological processes for spheroid sectioning necessitate exceptional precautions in handling, as sample tends to easily collapse or fracture. Acquisition of high-resolution images from spheroids is another concern because of the size and spherical shape. For nanomedicine penetration assessments in spheroids, it is sometimes difficult for compare results from different studies duo to completely different models applied. For instance, the fluorescence signal of the NP is measured in one study and by drug or release agent in another case [22] It should be consider that the penetration depth of the NPs within the spheroid is not a key indication of its effectiveness. In the case that NP is a nanocarrier, the release of the payload and its penetration is furthermost significant [6]. Nonetheless, tracking the payloads of the NPs is further problematic, particularly for molecules that does not hve fluorescence properties. Establishment of using spheroids in preclinical analysis might lessen the quantity of compounds progressing to animal studies, thus lessening the quantities of animals used.

Co-culture or tri-culture models are used to mimic the in vivo situation and to evaluate the impacts of nearby non-tumor cells on 3D systems. The spheroid heterotypic culture is more challenging compared to the spheroid monoculture; therefore, the cell culture media optimization is one of the main features in spheroid maintenance for cultivation of different types of cells. The spatial association of the cells in a spheroid is one of key features of co-culturing to be considered. In this context, several researchers found that, in a heterotypic model of pancreatic cancer in which tumor cells co-cultured with stellate cells, the stellate cells form the outer shell of the spheroid whereas the cancer cells form the central mass [127–130]. Although this pattern was not observed in the results of Norberg et al*.*, in which opposite results were reported. Additional investigation is desired to confirm which outcomes accurately reveals the condition in vivo [52]. The dextran/chitosan scaffold can offer surface chemistry that induces 3D microtumors with physiologically related features to in vivo tumor including propagation, morphology, ECM deposition, hypoxic phenotype, and drug response [131]. Likewise, the gelatin-fibronectin coating of fibroblast facilitated HUVEC adhesion and effective establishment of a tri-culture spheroids composed of cancer cell, fibroblast, and HUVEC [132]. Furthermore, gelatin-methacrylate and hyaluronan-methacrylate based in-air and photo-crosslinked microgels enabled formation of a heterotypic 3D spheroids of osteoblast and prostate cancer cells [133].

As mentioned previously, spheroids produced by co-culturing multiple cell types have much greater morphological complexity than simpler spheroids and 2D cultures, and multiparametric analysis will be required to investigate and quantify the cell response the treatment. Based on the published data, the presence of even 30% stromal cells resulted in the expression of ECM components such as fibronectin and collagen across the whole volume of heterospheroids. This is important as stromal microenvironment strongly affects the uptake of NPs and increased stiffness caused by augmented ECM deposition in tumor tissue as a physical barrier preventing intratumoral drug penetration. The analysis of NPs penetration and uptake in the function of stromal content provides an important evaluation parameter of therapeutic delivery systems and allows better optimizing of NPs design for in vivo biodistribution studies. This is particularly essential for drug penetration assessments as these models closely mimic the in vivo environment, consequently, can more efficiently predict the drug effects and delivery mechanisms. Moreover, in vivo translation of the spheroid model was shown to significantly improve subcutaneous xenograft model of cancer by reproducing stroma-rich tumors with rather thick ECM architecture recapitulating clinical tumors. In addition, application of these models can reduce costly investments related to the clinical examinations [10]. It should be also noted that not all tumor cell lines can be cultivated to form spheroids. Currently, formation of heterotypic and monotypic spheroid is restricted to a few specific cell types, which has been reviewed by K.A. Fitzgerald et al*.* [134]. Application of co-culture spheroids can increase the complexity and consistency of spheroid models to reach greater accuracy in drug assessments. Nevertheless, in co-culture spheroids, quantitative assessments of specific cell type are frequently problematic. In this case, it is beneficial to label each cell type using cell tracker stains or tagging the cells with fluorescently expressed proteins such as GFP although it necessitates extra transfection steps before spheroid establishment. Likewise, flow cytometric analysis of harvested spheroids would require a large sample size of spheroids of at least 10,000 cells [120].

The co-cultured spheroid usually lacks physiological flow, which is critical for distribution and penetration analysis of nanodrugs. Using the microfluidic systems or spheroid-on-a-chip platforms might restore the physiological flow to improve infiltration and successive biodistribution of drug [132]. Protocols for co-culture spheroid fabrication is very different and depend on the cell type, cell number, method, type of scaffold used, degree of scaffold, and etc. For this reason, it is essential to establish standardized methodologies for spheroid formation for the alignment of data from diverse labs. The challenges associated with spheroids can be additionally lengthened, nonetheless the point that requires superior attention is an urgent need for establishing the standardized accepted protocol for spheroid and other 3D cell cultures models such as co-culture spheroid and organoid [132]. In the case of nanomedicine, an accurate analysis of nanoparticle uptake and/or localization would be of excessive value. However, their tridimensionality also causes a struggle in computational image analysis and visualization as the complex topology and the thickness of spheroids, make image analysis challenging, and are incompatible with most automated imaging systems due to low light penetration and absorption across the multi-layered structures. Regarding nanodrugs evaluations, physicochemical features of the nanoformulation along with characteristics of different spheroid models need to be reconsidered. Suitable nanodrug evaluation protocols need to be available mentioning the most appropriate spheroid platform for a specific class of nanodrugs to assess their possible toxic effects.

Finally, patient-derived spheroids or organoids may provide robust preclinical drug-screening platforms to identify most effective personalized therapeutic option for cancer patients. These spheroids can offer valuable information about individual tumors due to recapitulating the original tumor characteristics [135, 136].

## Data Availability

Not applicable.

## Abbreviations

2D: Two-dimensional
3D: Three-dimensional
4T1: Mouse breast cancer
A2780: Human ovarian cancer cells
A549: Human lung cancer cells
AF555Au@1: Heparinoid-capped fluorescent AuNPs
andadult: Primary human dermal fibroblasts
APC: Antigen-presenting cells
a-SMA: Smooth muscle actin
AsPC-1: Human pancreatic cancer cell line
Au@GO: Gold NP-functionalized graphene oxide
B16F1: Murine melanoma cell line
BBB: Blood Brain Barrier
BCAFs: Fibroblast cells: human breast cancer-associated fibroblasts
BeWo: Human placental choriocarcinoma
Bio-PANPs: Biotin-conjugated pullulan acetate nanoparticles
BPAE: Bovine-pulmonary arterial endothelial
BPD: Benzoporphyrin derivative
CAFs: Cancer associated-fibroblasts
CAOV3: Human ovarian cancer cell line
CaOV3CisR: Cisplatin-resistant CaOV3 cell line
CCD-1059-SK: Human fibroblast cell line
CdTe-COOH: CdTe surface-functionalized with carboxylate moieties
Cmab-GNR: Cetuximab-anchored gold nanorod
DDC: Diethyldithiocarbamate
DMAEMA: N,N-dimethyl amino ethyl methacrylate
Dox: Doxorubicin
ECM: Extracellular matrix
ECs: Endothelial cells
EPR: Enhanced permeability and retention
EV: Extra vesicle
FaDu: Human pharynx squamous cell carcinoma
FAP: Fibroblast Activation Protein
GM-CSF: Granulocyte-macrophage colony-stimulating factor
GNP: Gold nanoparticle
H69AR: Human small cell lung cancer cell line
hBM-MSCs: Human bone-marrow derived MSCs
HBO: Hyperbaric oxygen
HCT116: Human colorectal carcinoma
HDLNPs: High-density lipoprotein NPs
hDPSC: Human Dental Pulp Stem Cells
HIF: Human intestinal fibroblast
HMF: Human mammary fibroblasts
HNSCC: Head and neck squamous cell carcinoma
HOF: Human ovarian fibroblasts
hpAs: Human primary astrocytes
hpBECs: Human primary brain endothelial cells
HPMC: Human primary mesothelial cells
hpPs: Human primary pericytes
hSkMC: Primary human skeletal muscle cells
HUVEC: Human umbilical vein endothelial cells
HVMF: Primary human villous mesenchymal fibroblasts
ICG: Indocyanine green
LCP-NPs: Lipid-coated calcium phosphate NPs
LS: Neuroblastoma cell line
LSFM: Light Sheet Fluorescence Microscopy
M2-M: M2-like macrophages
Mab-IR700: MRP1-targeted antibody-IR700 structure
matrigel: Gelatin methacryloyl
MCF-7: Human breast adenocarcinoma cells
MDA-MB-231: Triple negative breast cancer derived cell line
MeWo: Granular fibroblasts, derived from human melanoma
MIAPaCa-2: Human pancreatic ductal adenocarcinoma cell line
monocyte: Primary human monocyte
MPG: Cell penetrating peptide
MPG: Penetrating peptide
MRC-5: Human fetal lung fibroblasts
MSCs: Mesenchymal stem cells
nanoMOFs: Nanoscale iron carboxylate metal–organic frameworks
nano-TPP: Nanoencapsulated meso-tetraphenyl porphyrin
NE: Neutrophil elastase
NE-LMP: Chimeric cell membrane proteins and membrane-bound elastase
NHLF: Human Lung Fibroblasts
NIH-3T3: Mouse embryonic fibroblast
NIR-PIT: Near infrared photoimmunotherapy
NP: Nanoparticle
Nut3a: Chemotherapeutic Nutlin-3a
OA: Oleanolic acid
PA: Photoacoustic
Pan02: Murine model of pancreatic cancer
Panc-1: Pancreatic carcinoma of ductal cell
PC-3: Human prostate cancer cell line
PCAFs: Primary pancreatic cancer-associated fibroblasts
PDAC: Pancreatic ductal adenocarcinoma
PDA-ICG-DOX: Polydopamine nanoparticles loaded with indocyanine green-doxorubicin combinations
PD-L1: Programmed death ligand 1
PDT: Photodynamic therapies
PICs: Cetuximab photoimmunoconjugates
PIT: Antibody-targeted PDT
PSCs: Human immortalized pancreatic stellate cells
PT: Photothermal
PTM: Photothermal microscopy
PTT: Photothermal therapy
PTX: Paclitaxel
PTX/CALP: PTX-loaded corosolic acid liposomes
RAW264.7: Mouse monocyte macrophages
RB: Rose Bengal
RG2: Rat glioblastoma cell line
SDT: Sonodynamic therapy
SKOV-3: Human ovarian cancer cell line with epithelial-like morphology
SLN-Mito: Mitoxantrone-loaded solid tumor NPs
TAMs: Tumor associate macrophages
THP-1: Immortalized human monocytes
TIFP: Tumor interstitial fluid pressure
TME: Tumor microenvironment
ULA: Ultra-low attachment
UM-SCC-1: Head and neck cancer cell line
UMUC3: Human bladder cancer
Visudyne: Clinically approved liposomal formulation of benzoporphyrin derivative
WI-38: Human embryonal lung fibroblast cell line
